# Syndecan-4 Is a Key Facilitator of the SARS-CoV-2 Delta Variant’s Superior Transmission

**DOI:** 10.3390/ijms23020796

**Published:** 2022-01-12

**Authors:** Anett Hudák, Gábor Veres, Annamária Letoha, László Szilák, Tamás Letoha

**Affiliations:** 1 Pharmacoidea Ltd., 6726 Szeged, Hungary; anett.hudak@pharmacoidea.eu (A.H.); gabor.veres@pharmacoidea.eu (G.V.); labor@pharmacoidea.eu (L.S.); 2 Albert Szent-Györgyi Clinical Center, Department of Medicine, Faculty of Medicine, University of Szeged, 6720 Szeged, Hungary; letohadr@gmail.com

**Keywords:** SARS-CoV-2, Delta variant, viral transmission, cellular entry, syndecan, heparan sulfate proteoglycans

## Abstract

Emerging SARS-CoV-2 variants pose threats to vaccination campaigns against COVID-19. Being more transmissible than the original virus, the SARS-CoV-2 B.1.617 lineage, named the Delta variant, swept through the world in 2021. The mutations in the Delta’s spike protein shift the protein towards a net positive electrostatic potential. To understand the key molecular drivers of the Delta infection, we investigate the cellular uptake of the Delta spike protein and Delta spike-bearing SARS-CoV-2 pseudoviruses. Specific in vitro modification of ACE2 and syndecan expression enabled us to demonstrate that syndecan-4, the syndecan isoform abundant in the lung, enhances the transmission of the Delta variant by attaching its mutated spike glycoprotein and facilitating its cellular entry. Compared to the wild-type spike, the Delta one shows a higher affinity towards heparan sulfate proteoglycans than towards ACE2. In addition to attachment to the polyanionic heparan sulfate chains, the Delta spike’s molecular interactions with syndecan-4 also involve syndecan-4’s cell-binding domain that mediates cell-to-cell adhesion. Regardless of the complexity of these interactions, exogenously added heparin blocks Delta’s cellular entry as efficiently as syndecan-4 knockdown. Therefore, a profound understanding of the molecular mechanisms underlying Delta infections enables the development of molecularly targeted yet simple strategies to reduce the Delta variant’s spread.

## 1. Introduction

The pandemic caused by the severe acute respiratory syndrome coronavirus 2 (SARS-CoV-2) took the world by storm [1]. Unlike any other pandemic of modern times, SARS-CoV-2 and the related coronavirus disease 2019 (COVID-19) caused widespread panic in societies [2]. Various non-pharmaceutical interventions (NPIs) were utilized worldwide to prevent viral spread. Lockdowns, school closures, and travel bans were among the many NPIs adopted, all exerting limited effect on SARS-CoV-2 transmission [3,4]. The introduction of new and highly efficient vaccines against COVID-19, on the other hand, reduced the prevalence of severe COVID-19 cases worldwide [5]. However, the emergence of new variants poses a threat to the seemingly successful and efficient mass vaccination efforts against COVID-19 [6]. Named as a “Variant of Concern” by the WHO, Delta is a highly contagious SARS-CoV-2 mutant, first identified in India last year [7]. The SARS-CoV-2 Delta variant, also known as lineage B.1.617.2, or the Indian variant, swept rapidly through India and the United Kingdom, then reaching the United States, thus becoming the predominant variant worldwide (and recently being taken over by the emerging Omicron variant, now accounting for the majority of COVID-19 cases) [8,9]. The Delta variant has several mutations in its spike protein, leading to increased transmissibility and the ability to escape neutralizing antibodies triggered by the original SARS-CoV-2 strain [10,11,12]. The Delta’s mutations result in multiple replacements of neutral or negatively charged amino acids with positively charged ones [13]. On the other hand, the spike protein’s shift towards a positive electrostatic potential does not lead to increased affinity towards ACE2, a cell surface protease widely recognized as the main cell entry receptor for SARS-CoV-2 [14,15,16,17]. A recent study showed that mutating the intracellular domain of ACE2 does not affect the intracellular entry of SARS-CoV-2, suggesting that the internalization of the virus is driven by ACE2 independent receptors [18]. According to these findings, the virus attaches to ACE2; however, the membrane engulfment and subsequent internalization is mediated by other, ACE2-independent endocytosis receptors.

It has been well established that SARS-CoV-2 exploits several receptors to enter the cells [19]. Heparan sulfate proteoglycans (HSPGs) offer highly versatile polyanionic glycosaminoglycan (GAG) chains for SARS-CoV-2 to attach to the cell surface [20,21,22,23,24]. We have previously shown that syndecans (SDCs), the transmembrane family of HSPGs, facilitate SARS-CoV-2’s cellular internalization [25]. Among SDCs, syndecan-4 (SDC4), an SDC isoform highly abundant in the lung (see the BioGPS gene expression database, http://biogps.org, accessed on 9 November 2021, for more details), mediated SARS-CoV-2’s cellular entry the most [25,26,27,28]. The spike protein’s heparin-binding core motif (PRRAR) has a paramount influence on the interaction with SDCs, yet SARS-CoV-2’s interactions with SDCs are more complex than just attachment to SDCs heparan sulfate (HS) side chains. Considering the Delta spike’s significant shift towards positive electrostatic potential and the role of SDCs in mediating the internalization of a wide array of ligands (with an affinity towards HS), in this paper we analyze the cellular interaction and internalization of the Delta variant’s spike protein. The targeted in vitro manipulation of ACE2 and SDC expression enables us to understand the main molecular drivers responsible for Delta’s efficient cellular entry. The results obtained in our ACE2 and SDC-specific cellular models demonstrate the SDC4-mediated enhancement of the Delta variant’s superior transmissibility.

## 2. Results

### 2.1. Effect of ACE2 Overexpression on the Cellular Internalization of the Spike Proteins

**Figure 1:**
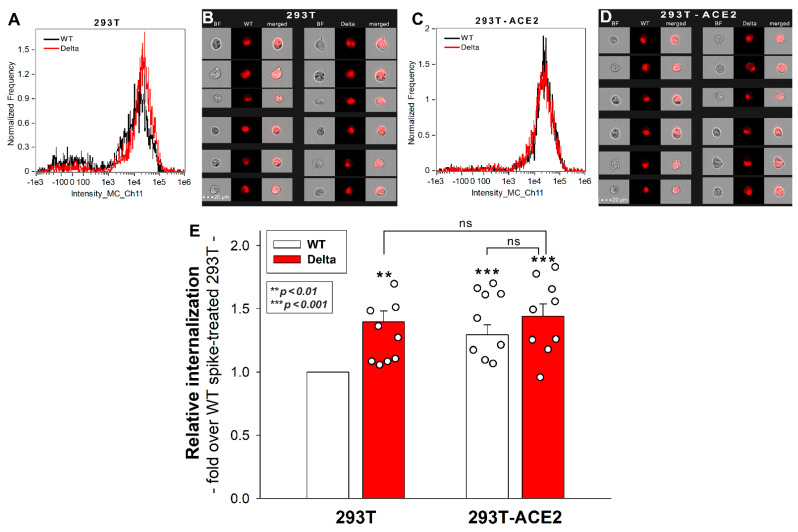
**Cellular uptake of WT and Delta spike into 293T and ACE2 overexpressing 293T-ACE2 cells**. The cells were incubated with WT and Delta spike proteins for 4 h at 37 °C. After incubation, the cells were washed, trypsinized, fixed, permeabilized and treated with AF 647-labeled antibodies specific for the His-tag of the recombinant spike proteins. Cellular uptake was then analyzed with imaging flow cytometry. (**A**–**D**) Representative flow cytometry histograms (**A**,**C**), brightfield (BF) and fluorescent cellular images (**B**,**D**) showing the intracellular fluorescence of 293T and 293T-ACE2 cells treated with WT or Delta spike proteins. Scale bar = 20 μm. (**E**) Detected fluorescence intensities were normalized to WT spike-treated 293T cells as standards. The bars represent the mean + SEM of nine independent experiments. Experimental data are presented as dots. Statistical significance was assessed with ANOVA. ** *p* < 0.05 vs. standards; *** *p* < 0.01 vs. standards; ns: not significant.

The cell surface ACE2 has been widely recognized as the primary cell entry receptor for SARS-CoV-2 [16,17]. Possessing an ACE2 specific receptor-binding domain (RBD), the spike protein plays a crucial role in initiating the attachment to the ACE2 receptor [16]. To study the involvement of ACE2 in the cellular entry of the wild-type (WT) SARS-CoV-2 spike and its Delta variant, ACE2 overexpressing 293T-ACE2 cells, along with standard 293T cells were treated with the WT spike protein and its Delta variant for 4 h at 37 °C at a concentration of 50 nM. Cellular uptake was then detected by incubating the spike-treated, fixed and permeabilized cells with Alexa Fluor 647 (AF 647) labeled antibody specific for the N-terminal His-tag of the recombinant WT and Delta spike proteins. Before the imaging flow cytometry analyses, surface-attached spikes were removed with trypsinization, according to the method described by Nakase et al. [29,30]. Hence, the imaging flow cytometer (Amnis FlowSight) only measured the internalized spike proteins [25]. (As shown in Supplementary Figure S1, trypsinization efficiently removes extracellularly attached fluorescent proteins.) Imaging flow cytometry analyses revealed that the Delta spike is internalized by 293T cells more efficiently than the WT spike protein (Figure 1A,B,E). Overexpression of ACE2 in 293T-ACE2 cells increased the internalization of the WT spike. Compared to Delta-treated 293T cells, Delta uptake into 293T-ACE cells remained almost unchanged, suggesting that Delta’s cellular entry is less dependent on ACE2 than WT spike’s (Figure 1E). Incubating the cells with the fluorescently labeled anti-His tag antibodies without spike pretreatment did not induce any difference, showing that no specific binding influenced the obtained results (Supplementary Figure S2). Additionally, neither spike protein affected cellular viability at the applied concentration of 50 nM (Supplementary Figure S3).

### 2.2. 293T-ACE2 Cells Exhibit Increased ACE2 Yet Reduced HS and SDC Expression

**Figure 2:**
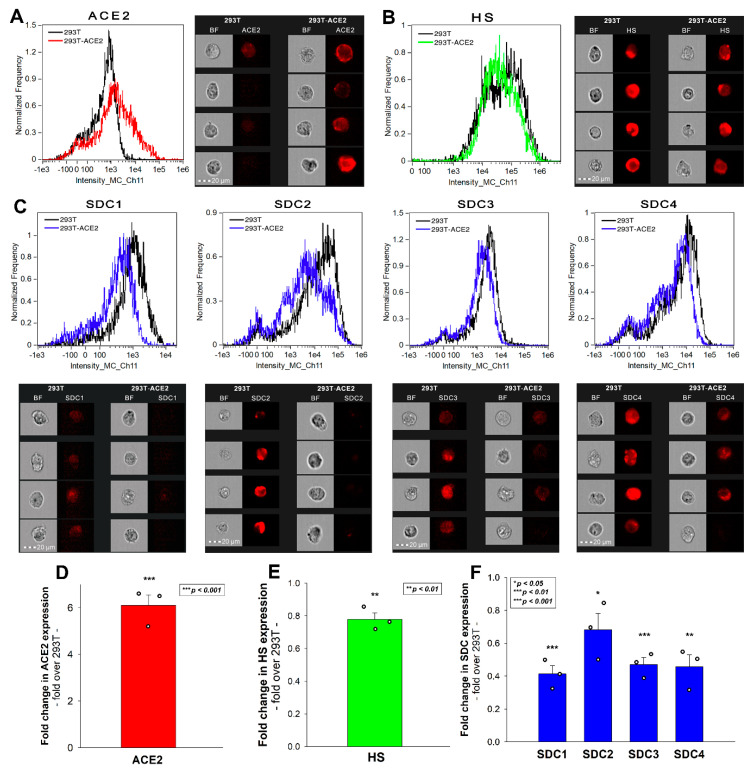
**ACE2, HS, and SDC expression of 293T and 293T-ACE2 cells.** ACE2, HS, and SDC expression of 293T and 293T-ACE2 cells was measured with imaging flow cytometry by using fluorescently labeled specific antibodies. (**A**,**B**) ACE2 and HS expression profile of 293T and 293T-ACE2 cells. The representative flow cytometry histograms and cellular images show the ACE2 and HS expression of 293T and 293T-ACE2 cells treated with the fluorescent ACE2 and HS antibodies. Scale bar = 20 μm. (**C**) SDC expression profile of 293T and 293T-ACE2 cells. The representative flow cytometry histograms and fluorescent cellular images show the SDC expression of 293T cells and 293T-ACE2 cells treated with the APC-labeled respective SDC antibodies. Scale bar = 20 μm. (**D**–**F**) Detected ACE2, HS and SDC expression measures were normalized to those of 293T cells as standards. The bars represent the mean ± SEM of three independent experiments (data are represented as dots). Statistical significance vs. standards was assessed with ANOVA. * *p* < 0.05; ** *p* < 0.01; *** *p* < 0.001.

Simultaneously to the spike uptake studies, we also measured the expression of ACE2 and HS in both 293T and 293T-ACE2 cells. Compared to 239T, increased ACE2 expression of 293T-ACE2 cells (i.e., ACE2 expression was about six times that of 293T cells) was associated with significantly lower (~80%) cell surface HS expression (Figure 2A,B,D,E). To examine whether the expression of SDCs was also affected by ACE2 overexpression, we also measured SDC expression with flow cytometry. These analyses revealed significantly decreased the expression of all four SDC isoforms (i.e., SDC1–4) in ACE2 overexpressing ACE2-293T cells (Figure 2C,F). Studies with respective isotype controls revealed no difference in isotype control-treated 293T and 293T-ACE2 cells (Supplementary Figure S4). Although the detailed molecular mechanism of ACE2 affecting membrane HS and SDC expression is still not fully understood (the lipid raft-associated ACE2 might disrupt the expression of lipid raft-associated HSPGs, including SDCs), this serendipity led to a valuable finding. In the case of the Delta spike, overexpression of ACE2 cannot compensate for the reduced HS and SDC content of 293T-ACE2 cells, highlighting the paramount importance of SDCs and polyanionic cell surface HSPGs in the interaction with the Delta spike, enriched in positively charged amino acids. Thus, the Delta spike protein exhibits a higher affinity towards HSPGs’ HS chains than towards ACE2.

### 2.3. Effect of ACE2 and Proteoglycan Inhibition on Cellular Uptake

SARS-CoV-2 infection is initiated by the virus’ attachment to the host cells’ ACE2, while cellular entry is mediated by the host cell’s endocytic pathways [16,18]. Endocytosis is an active process orchestrated by signaling pathways stimulated by the ligand’s extracellular attachment to specific endocytic receptors [31]. A recent cellular study showed that the ACE2-mediated signaling is not essential for SARS-CoV-2’s cellular entry, suggesting that endocytosis might be driven via ACE2-independent endocytic receptors [18]. DX600 is a competitive peptide inhibitor of ACE2 that only modestly inhibits the cellular entry of SARS-CoV-2 (and -1), suggesting that ACE2’s enzymatic activity is not essential for viral endocytosis [25,32,33]. On the other hand, heparin reduces SARS-CoV-1 and -2 infection very efficiently by directly competing with viral binding to host HSPGs [22,34,35].

**Figure 3:**
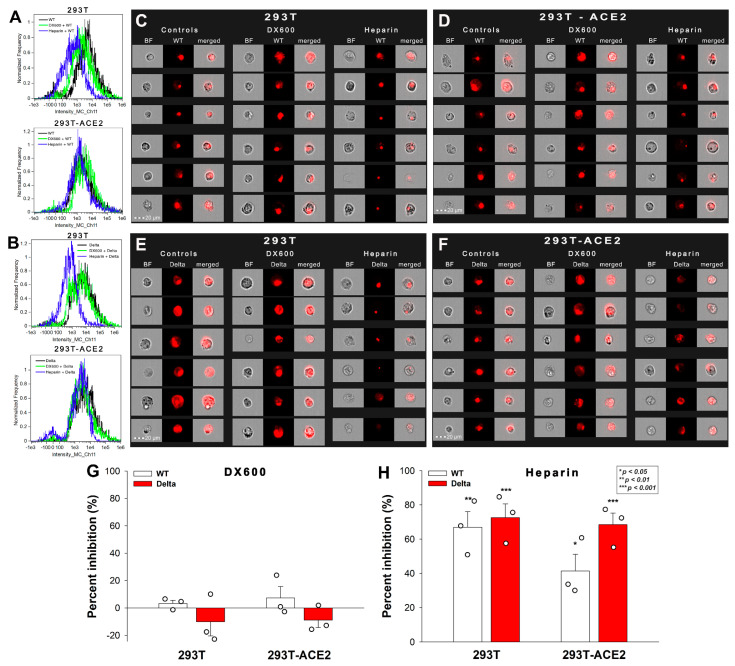
**Effect of ACE2 and proteoglycan inhibition on cellular uptake.** 293T and 293T-ACE2 cells, preincubated with or without heparin (200 ug/mL) or DX600 (10 uM), were treated with the WT or Delta spike proteins. (**A**–**F**) Representative flow cytometry histograms (**A**,**B**) and fluorescent cellular images (**C**–**F**) show the fluorescence of 293T and 293T-ACE2 cells treated with either the WT or Delta spike proteins in the presence or absence of the inhibitors. Scale bar = 20 μm. (**G**,**H**) The effect of the inhibitors expressed as percent inhibition, calculated with the following formula: ((X − Y)/X) × 100, where X is the fluorescence intensity obtained on cells treated with the spike proteins in the absence of the inhibitor, and Y is the fluorescence intensity obtained on cells treated with proteins in the presence of the inhibitor. The bars represent the mean ± SEM of three independent experiments (experimental data are represented as dots). Statistical significance vs. standards was assessed with ANOVA. * *p* < 0.05; ** *p* < 0.01; *** *p* < 0.001.

To further explore the role of ACE2 and HSPGs in the cellular uptake of the spike proteins, we studied the effect of the two competitive inhibitors of ACE2 and HSPGs (i.e., DX600 and heparin) on the cellular uptake of WT and Delta spike. Thus, 293T-ACE2 and standard 293T cells, preincubated with or without DX600 or heparin, were treated with the WT spike protein and its Delta variant for 4 h at 37 °C at a concentration of 50 nM. Unlike DX600, which left the cellular uptake of the spike proteins unaffected, heparin reduced the WT and Delta spike uptake in 293T and 293T-ACE2 cells, as shown by data obtained with imaging flow cytometry (Figure 3A–H). Heparin’s effect on lowering the cellular uptake of Delta was slightly higher than on WT spike, suggesting the increased importance of polyanionic HS chains in the interactions with the more positively charged Delta spike (Figure 3A–F,H). On the other hand, heparin’s effect on reducing the cellular uptake of WT spike into 293T-ACE2 cells was smaller than on 293T, showing that increased ACE2 expression leads to increased ACE2-dependent interactions with WT spike.

These results also showed that, compared to WT spike, Delta has a greater affinity towards HSPGs, than towards ACE2. The observation that ACE2 activity is not required for the cellular entry of either the WT or the Delta spike proteins suggests that ACE2 functions as a binding site instead of a receptor actively driving the mechanism of viral endocytosis.

### 2.4. SDC4 Enhances the Cellular Uptake of the Delta Spike Protein

**Figure 4:**
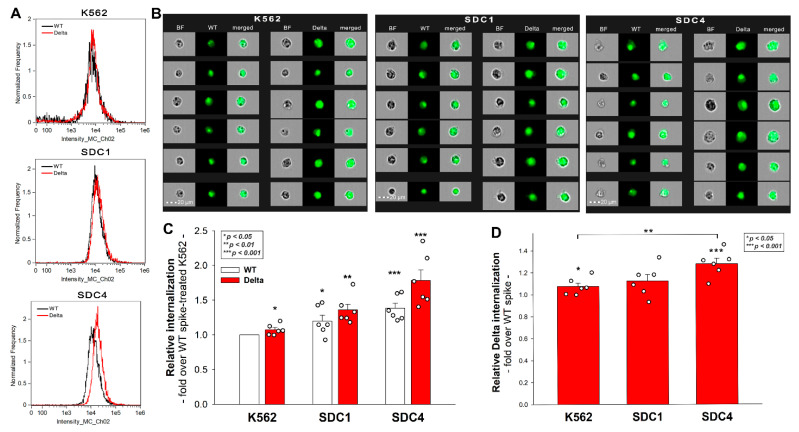
**Cellular uptake of the WT spike and its Delta variant into WT K562 cells and SDC1, 4 transfectants.** WT K562 cells, SDC1 and SDC4 transfectants were treated with the WT spike and its Delta variant. The cellular uptake was then analyzed with imaging flow cytometry by using fluorescently labeled specific antibodies against the His-tag of the recombinant spikes. (**A**,**B**) Representative flow cytometry histograms and cellular images show the intracellular fluorescence of WT K562 cells, SDC1 and SDC4 transfectants treated with either of the spike proteins. Scale bar = 20 μm. (**C**) Detected fluorescence intensities normalized to WT-spike-treated K562 cells as standards. The bars represent the mean + SEM of six independent experiments (data are represented as dots). Statistical significance vs. standards was assessed with ANOVA. * *p* < 0.05; ** *p* < 0.01; *** *p* < 0.001. (**D**) Detected fluorescence intensities normalized to WT-spike-treated cells as standards. The bars represent the mean + SEM of six independent experiments (data are represented as dots). Statistical significance vs. standards was assessed with ANOVA. * *p* < 0.05; ** *p* < 0.01; *** *p* < 0.001.

Our latest SARS-CoV-2 study showed that SDCs, especially SDC4 enriched in the lung, facilitate SARS-CoV-2’s entry into the cells by attaching the S1 subunit of the spike protein [25]. Other research groups also implied the role of SDC1 in SARS-CoV-2 infection [24,36,37,38]. Thus, SDC1 and SDC4 transfectants, created in human myeloid K562 cells, were incubated with WT and Delta spike proteins. Due to their modest HSPG expression (except for minor amounts of endogenous betaglycan, K562 cells are devoid of HSPGs), K562 cells offer ideal cellular models to study the contribution of SDCs to the cellular uptake of potential ligands without the interfering effects of other HSPGs [25,39,40]. As HS has already been established as one of the key binding sites for SARS-CoV-2 [20,21,22,23], stable SDC transfectants created in K562 cells were standardized according to their HS content (Supplementary Figure S5) [39,40]. As reported previously, SDC transfection did not induce statistically significant changes in ACE2 expression [25]. Thus, SDC1 and SDC4 transfectants with an equal amount of HS expression were selected and, along with WT K562 cells, treated with WT and Delta spike proteins at a concentration of 50 nM for 4 h at 37 °C. Compared to WT K562 cells lacking SDC1 and 4 (thus, WT K562 cells could be considered as SDC1 or SDC4 KO lines), SDC1 or SDC4 overexpression increased the cellular uptake of the WT spike and its Delta variant (Figure 4A–C). SDC overexpression further increased the internalization efficacy of the Delta spike, especially SDC4, which increased the difference between the cellular uptake of the WT spike and Delta the most (Figure 4C,D).

Control studies with WT K562 cells and SDC transfectants treated with FITC-labeled a-His at 0 °C showed the efficacy of trypsinization in removing extracellularly attached proteins (Supplementary Figure S6). As shown in Supplementary Figure S7, neither the WT nor the Delta spike affected cell viability after 4 h of incubation at the applied concentration.

### 2.5. SDC4 Enhances the Cellular Uptake of the Delta PSV

**Figure 5:**
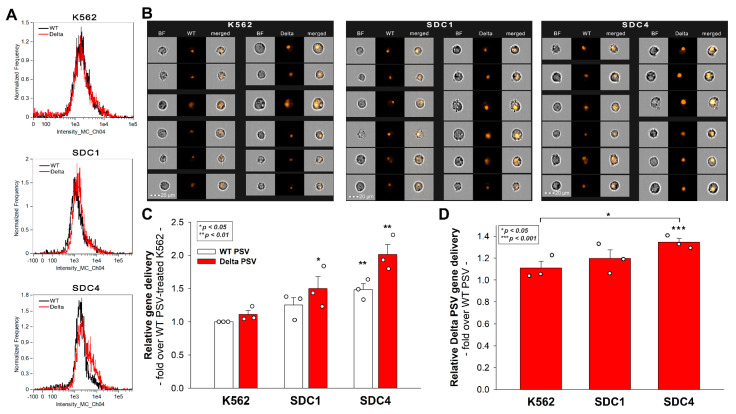
**PSV-mediated gene transduction into WT K562 cells, SDC1 and SDC4 transfectants.** The cells were incubated with SARS-CoV-2 PSV-RFP (either the WT or the Delta variant). RFP expression was analyzed 72 h later with imaging flow cytometry. (**A**) Representative flow cytometry histograms showing RFP fluorescence of WT K562 cells, SDC1 and SDC4 transfectants incubated with either the WT or the Delta SARS-CoV-2 PSV. (**B**) Cellular images of SARS-CoV-2 PSV-treated WT K562 cells and SDC transfectants as detected with imaging flow cytometry. Scale bar = 20 μm. (**C**) Detected cellular RFP intensities normalized to WT PSV-treated WT K562 cells as standards. The bars represent the mean + SEM of three independent experiments (data are represented as dots). Statistical significance vs. standards was assessed with ANOVA. * *p* < 0.05; ** *p* < 0.01. (**D**) Detected RFP intensities normalized to WT PSV-treated cells as standards. The bars represent the mean ± SEM of three independent experiments (data are represented as dots). Statistical significance vs. standards was assessed with ANOVA. * *p* < 0.05; *** *p* < 0.001.

To further seek evidence of the involvement of SDCs in the cellular entry of the Delta variant, SDC1 and SDC4 transfectants, along with WT K562 cells (lacking SDC1 and SDC4), were incubated with SARS-CoV-2 PSVs, recombinant pseudotyped lentiviral particles carrying either the WT or the Delta spike and encoding the red fluorescent protein (RFP) as a reporter gene [41,42,43,44]. Studies with SARS-CoV-2 PSVs delivered similar results as those with the spike proteins. Namely, both SDC1 and SDC4 increased PSV-mediated gene (i.e., RFP transduction) either for the WT or Delta PSVs. However, the rate of SDC-mediated increase was higher for the Delta PSV (Figure 5A–D). The most efficient transduction was achieved with Delta PSV on SDC4 transfectants, showing that SDC4 enhances Delta cellular entry the most (Figure 5C,D).

### 2.6. Exploring the Delta Spike’s Interactions with the SDC4 Ectodomain

**Figure 6:**
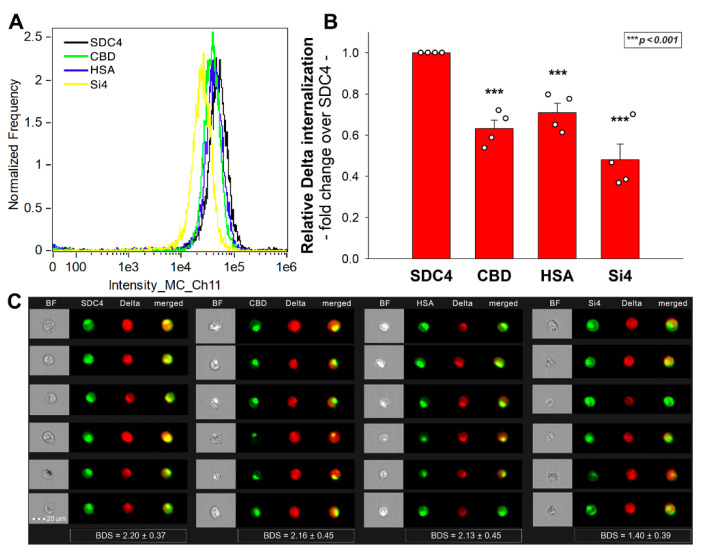
**Cellular uptake of the WT and Delta spike proteins into SDC4 deletion mutants.** WT SDC4 transfectants and CBD, HSA and Si4 mutants (all tagged with GFP) were treated with the Delta spike. The cellular uptake was then analyzed with imaging flow cytometry by using fluorescently labeled specific antibodies against the His-tag of the recombinant spike proteins. (**A**) Representative flow cytometry histograms showing the intracellular fluorescence of WT SDC4 transfectants, CBD, HSA and Si4 mutants treated with the Delta spike protein (along with specific fluorescent antibodies). Scale bar = 20 μm. (**B**) Detected fluorescence intensities were normalized Delta spike-treated WT SDC4 transfectants as standards. The bars represent the mean ± SEM of four independent experiments (experimental data are represented as dots). Statistical significance vs. standards was assessed with ANOVA. *** *p* < 0.001. (**C**) Cellular images showing the intracellular fluorescence of WT SDC4 and CBD, HSA and Si4 mutants treated with the Delta spike protein. Scale bar = 20 μm. The indicated BDS values of SARS-CoV-2 and SDCs represent the mean + SEM of four independent experiments.

To explore the molecular drivers of SDC4’s interaction with the Delta spike, a Delta spike protein was added to K562 transfectants expressing SDC4 mutants with truncated ectodomains [25]. Among the applied SDC4 mutants, Si4 lacks the whole SDC4 ectodomain except for the signal sequence, while the ectodomain of CBD is made of only the cell-binding domain (CBD) of SDC4. The extracellular domain of HSA mutants compromises only the HS attachment site (HSA) with the attached HS chains, but no CBD. These SDC4 structural mutants and the WT SDC4 were tagged GFP and expressed in WT K562 cells. Transfectants with similar SDC4 expression were selected with flow cytometry and treated with the Delta spike protein (at a concentration of 50 nM). After 4 h of incubation at 37 °C, the cellular uptake was detected by incubating the Delta spike-treated, fixed and permeabilized cells with fluorescently (AF 647) labeled antibody specific for the His-tag of the recombinant Delta spike. Extracellularly attached Delta spike proteins were removed with trypsinization, so only the internalized Delta spike proteins were measured (see control studies in Supplementary Figure S8) [25,29,30]. Compared to WT SDC4, all structural mutants exhibited significantly decreased Delta spike internalization (Figure 6A–C). Among them, Si4 demonstrated the highest decrease in Delta spike uptake, suggesting that simultaneous removal of SDC4’s CBD and HS chains induces the most significant reduction in Delta spike entry. The significantly decreased Delta spike uptake into CBD and HSA mutants also shows that both the CBD and the polyanionic HS chains are involved in the attachment of Delta spike protein. Thus, the interaction of the Delta spike protein with SDC4 extends beyond the HS chains and also involves SDC4’s CBD, mediating cell-to-cell attachment. The bright detail similarity (BDS) score of colocalization also showed a high degree of colocalization (generally, a BDS score of two or greater represents a high degree of overlap), especially in the case of SDC4 transfectant and the CBD and HSA mutants (Figure 6C).

The jointly shared internalization pathway of the CBD and HSA mutants and the Delta spike confirms that both the polyanionic HS chains and the CBD contain sequences the Delta can bind. Thus, Delta’s interactions with SDC4 extend beyond electrostatic interactions with the polyanionic HS chains.

### 2.7. ACE2, HS and SDC Expression Profile of Calu-3 Cells

In their very recent study, Arora et al. explored the internalization efficacy of the Delta spike variant on various cell lines [15]. In those studies, Caco-2 and Calu-3 cells, compared to 293T, exhibited a markedly increased cellular uptake of the Delta spike variant. Among Caco-2 and Calu-3, the increase in Delta cell entry was most pronounced in Calu-3 lung cells. According to the Human Protein Atlas (http://www.proteinatlas.org, accessed on 9 November 2021), ACE2 in Caco-2 cells is undetectable, while HEK cells (293T’s the parent cell line) show very modest ACE2 expression (Supplementary Table S1) [45]. In the case of SDCs, HEK and Caco-2 cells show similar expression profiles, except for SDC4, which is expressed about 30 times more in Caco-2 than in HEK cells [46]. To explore the HS-mediated internalization of the SARS-CoV-2 D614G mutant, Yue et al. measured the ACE2 and the HS expression of several cell lines, including 293T and Calu-3 [47]. According to their flow cytometric analyses, both 293T and Calu-3 express low levels of cell surface ACE2, while the HS expression of Calu-3 is lower [47].

**Figure 7:**
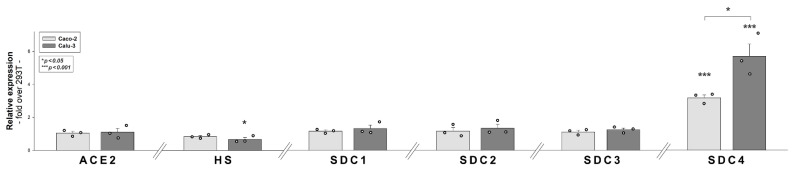
**Comparative expression analysis of ACE2, HS and SDCs in 293T, Caco-2 and Calu-3 cells.** ACE2, HS and SDC expression profile of the cell lines were analyzed with flow cytometry using fluorescently labeled antibodies. Detected expression levels were normalized to those of 293T cells. The bars represent the mean ± SEM of three independent experiments (data are represented as dots). Statistical significance vs. 293T expression levels was assessed with ANOVA. * *p* < 0.05; *** *p* < 0.001.

Considering the different cell entry efficacy of the Delta spike variant in 293T, Caco-2 and Calu-3 cells, along with the reported ACE2, HS and expression profile of these cell lines, we measured the ACE2, HS and SDC surface expression of 293T, Caco-2 and Calu-3 cells with flow cytometry using specific antibodies. Compared to 293T cells, Caco-2 and Calu-3 express similar amounts of ACE2, SDC1, SDC2 and SDC3 on their cell surface, but significantly (*p* < 0.001) increased amount of SDC4 (Figure 7 and Figure S9). Compared to 293T, Calu-3 cells also express significantly (*p* < 0.05) less HS, while the SDC4 expression of Calu-3 cells is also significantly (*p* < 0.05) higher than that of Caco-2 (Figure 7 and Figure S9).

### 2.8. SDC4 Knockdown Decreases Delta’s Internalization into Calu-3 Cells

**Figure 8:**
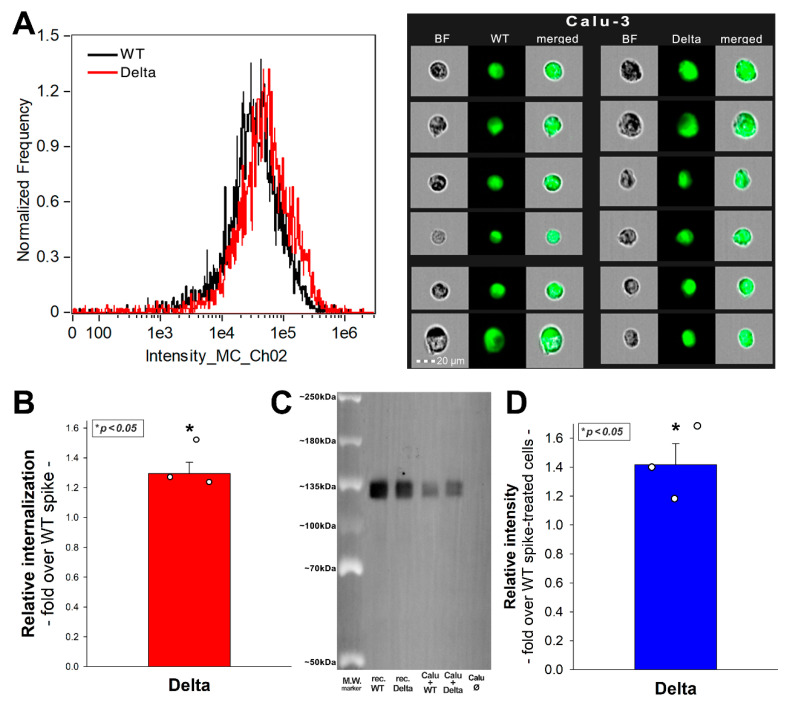
**Internalization of the WT and Delta spike into Calu-3 cells.** Calu-3 cells were treated with the WT spike and its Delta variant. The cellular uptake was then analyzed with imaging flow cytometry by using fluorescently labeled specific antibodies against the His-tag of the recombinant spike proteins. (**A**) Representative flow cytometry histograms and cellular images show the intracellular fluorescence of Calu-3 cells treated with either the WT or Delta spike. (**B**) Detected intracellular fluorescence levels normalized to those treated with the WT spike as standards. The bars represent the mean + SEM of three independent experiments. Statistical significance vs. 293T expression levels was assessed with ANOVA. * *p* < 0.05. (**C**) SDS-PAGE showing the WT or Delta spike immunoprecipitated with SDC4 antibodies from extracts of untreated control or spike-treated Calu-3 cells. Lanes 1–2: 0.5 ug of WT and Delta spike proteins; Lanes 3–4: immunoprecipitate of WT or Delta spike-treated Calu-3 cells; Lane 5: immunoprecipitate of untreated Calu-3. Standard protein size markers are indicated on the left. The signal of spike proteins was detected with UVITEC Alliance Q9 Advanced Imager, and the intensity of bands were analyzed with the NineAlliance^©^ software. (**D**) Detected band intensities were normalized to WT spike-treated Calu-3 cells as standard. The bars represent the mean + SEM of three independent experiments. Statistical significance vs. standards was assessed with ANOVA. * *p* < 0.05.

Considering our results on the superior ability of SDC4 to mediate the spike protein and its Delta variant, we progressed to study the cellular uptake of the spike proteins in Calu-3 cells showing relatively high SDC4 expression. Calu-3 cells were thus incubated with the WT and Delta spike proteins at a concentration of 50 nM for 4 h at 37 °C. Cellular uptake was then assessed with imaging flow cytometry. Trypsinization, described by Nakase et al., was applied to remove extracellularly attached spike proteins (see Supplementary Figure S10 showing the efficacy of trypsinization to remove extracellularly attached proteins) [29,30]. According to the obtained flow cytometry data, the Delta spike protein’s cellular internalization, compared to the WT spike, was about 1.35 times more efficient (Figure 8A,B). Co-immunoprecipitation studies showed that the Delta spike binds to SDC4 more efficiently than the WT spike (Figure 8C). As shown in Supplementary Figure S11, neither the WT nor the Delta spike proteins affected cell viability after 4 h of incubation at 50 nM.

**Figure 9:**
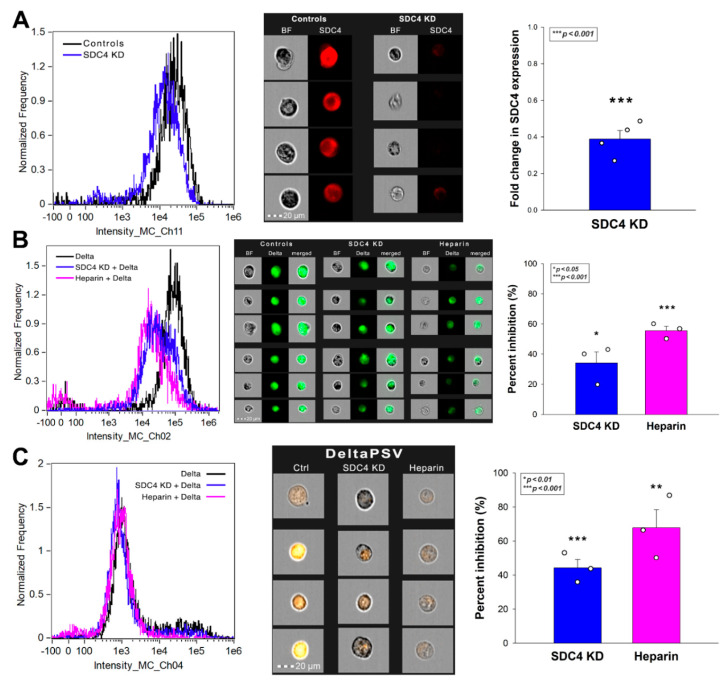
**Effect of SDC4 knockdown (KD) or GAG inhibition on Delta spike internalization into Calu-3 cells.** SDC4 KD in Calu-3 cells was performed using a lentiviral vector specific to human SDC4. (**A**) SDC4 expression levels were measured with imaging flow cytometry, as shown by the representative histograms and cellular images. Detected SDC4 levels of KD cells were normalized to WT Calu-3 cells as standards. The bars represent the mean + SEM of three independent experiments. Statistical significance vs. standards was assessed with ANOVA. * *p* < 0.05. (**B,C**) SDC4 KD and WT Calu-3 cells were treated with either the Delta spike protein (**B**) or the Delta PSV (**C**). For GAG inhibition, some of the WT Calu-3 cells were preincubated with heparin (200 ug/mL, 30 min before Delta treatment). Representative flow cytometry histograms and cellular images show the intracellular fluorescence of WT or SDC4 KD Calu-3 treated with Delta spike protein or PSV in the presence or absence of heparin. Scale bar = 20 μm. The effect of SDC4 KD or heparin treatment was expressed as percent inhibition, calculated with the following formula: ((X − Y)/X) × 100, where X is the fluorescence intensity obtained on controls treated with the Delta spike or Delta PSV without SDC4 KD or heparin, and Y is the fluorescence intensity obtained on cells receiving Delta spike or Delta PSV treatment, along with SDC4 KD or heparin. The bars represent the mean + SEM of three independent experiments. Statistical significance vs. controls was assessed with ANOVA. * *p* < 0.05; ** *p* < 0.01; *** *p* < 0.001.

The cellular uptake of the Delta spike could be significantly reduced with either SDC4 knockdown (KD) or heparin co-incubation (Figure 9A–C). Here, we have to note that as we could not achieve 100% KD of SDC4, as the efficacy of KD was around 60% (Figure 9A, Figures S12 and S13). Thus, a fold change of 0.6 in SDC4 expression exerted an uptake inhibition of about 34%, while heparin inhibited the Delta spike uptake with about 55% efficacy (Figure 9B). SDC4 KD or heparin co-incubation also decreased the gene transduction efficacy of the Delta PSV. Namely, heparin inhibited the Delta PSV-mediated gene delivery with ~68% efficacy, compared to the ~44% inhibition efficacy of SDC4 KD (Figure 9C).

## 3. Discussion

Emerging variants pose serious threats to vaccination campaigns against SARS-CoV-2 [48,49]. Variants of concern with increased transmissibility are starting to dominate the new COVID-19 case counts, hence causing widespread worry among experts [48]. First identified in India in October 2020, the SARS-CoV-2 B.1.617 lineage (named Delta variant) became the dominant strain worldwide in mid-2021 (before being taken over by the emerging Omicron in December 2021) [9,50,51,52,53]. According to scientific reports, the Delta variant is markedly more transmissible than the original SARS-CoV-2 strain [15,52,54]. The increased spread of the Delta variant is associated with an escape from antibodies that target non-RBD and RBD epitopes of the spike protein [52]. The spike protein of the Delta variant, responsible for attaching the virus to host cells, harbors several mutations, resulting in multiple replacements of neutral or negatively charged amino acids with positively charged ones [13]. Thus, Delta’s mutations shift the spike protein towards a net positive electrostatic potential. Arora et al. recently reported that Delta’s increased entry is not due to augmented ACE2 binding [15]. Therefore, Delta’s superior transmissibility should be attributed to interactions with ACE2-independent cellular entities. To examine the cellular entry of the Delta, first, we explored ACE2’s effect on the cellular internalization of the WT and Delta proteins. Although Delta could be taken up more efficiently by 293T cells, 293T-ACE2 cells with increased ACE2, but decreased HS and SDC expression failed to internalize the Delta spike at an increased rate. Although the suppression of SDCs and HSPGs due to ACE2 overexpression is not fully understood (as a lipid-raft associated glycoprotein, ACE2 might suppress the expression of other lipid-raft associated HSPGs, including SDCs), still, this unexpected serendipity enabled us to draw a valuable conclusion. Namely, that Delta spike exhibits a higher affinity towards polyanionic HSPGs and SDCs than towards ACE2.

Revealing the increased affinity of the Delta spike towards HSPGs, we proceeded to analyze the cellular uptake of the WT and Delta spikes in SDC1 and SDC4 specific cellular assays. Overexpressing SDC4 and SDC1 in K562 cells offers a great advantage: due to the lack of SDC1 and SDC4 expression, WT K562 cells can serve as a genuine SDC1 and 4 KD cell line. Utilizing SDC1 and SDC4 transfectants created in K562 cells revealed that SDCs increase the cellular uptake of the spike proteins, along with RFP-mediated gene delivery. Among SDCs, the lung abundant SDC4 further augmented the superior internalization efficacy of the Delta variant. As the applied SDC1 and SDC4 transfectants were standardized according to HS expression (SDC1 and SDC4 transfectants express similar amounts of HS), the superior efficacy of SDC4 in mediating Delta cell entry should also be attributed to HS-independent factors. Studies with SDC4 structural mutants confirmed the involvement of the cell-binding domain (CBD, mediating cell-to-cell attachment) in the interaction with the Delta variant, showing that Delta’s interactions with SDC4 extend beyond just electrostatic interactions with the polyanionic HS chains. This observation is also supported by the report of Arora et al., showing that cell lines with high SDC4 expression, irrespective of the amount of cell surface HS or ACE2, exhibit the most efficient cellular uptake of the Delta variant. Further uptake studies showed that the augmented transmissibility of the Delta variant could be decreased by reducing SDC4 expression, reinforcing the role of SDC4 in contributing to the superior cellular entry of the Delta variant.

In addition to the contribution of SDC4 to the superior transmissibility of the Delta variant, our in vitro studies helped to draw another significant conclusion. Namely, that ACE2 activity is not required for the efficient cellular translocation of the virus. The finding that ACE2 inhibition does not lead to reduced cellular uptake of the spike proteins shows that ACE2 rather serves as a docking site, while other endocytic receptors drive the endocytosis of the virus. In our case, SDCs.

In summary, our data obtained by fairly simple, yet highly specific cellular models highlight the significant role of SDC4 (and to a lesser extent, SDC1) in mediating the cellular internalization of the Delta variant. Studies on SDC4 structural mutants exposed the involvement of the CBD of the SDC4 ectodomain, suggesting that the Delta’s interactions with SDC4 extend beyond attachment to the HS chains. Regardless, exogenous heparin efficiently reduces the cellular entry of the Delta variant, showing that understanding the molecular drivers of the Delta variant’s superior transmission characteristics could lead to simple yet molecularly rational medical interventions blocking Delta infections. Further studies with virulent strains in clinically relevant in vivo models should confirm the therapeutic applicability of the current in vitro findings.

## 4. Materials and Methods

### 4.1. Recombinant Proteins and Pseudoviruses

The N-terminally His-tagged recombinant SARS-CoV-2 spike protein and its Delta variant were purchased from Sino Biological (Beijing, China, cat. no. 40589-V08B1-100 and 40589-V08B16-100), while the recombinant human SDC4 were obtained from R&D Systems (Minneapolis, MN, USA; cat. no. 2918-SD). SARS-CoV-2 pseudovirus-RFP (SARS-CoV-2 PSV-RFP), recombinant pseudotyped lentiviral particles bearing either the SARS-CoV-2’s WT spike protein or its Delta variant and encoding the red fluorescent protein (RFP), was purchased from GeneMedi (Shanghai, China; cat. no. GM-2019nCoV-PSV01 and GM-2019nCoV-PSV34).

### 4.2. ACE2 and SDC Constructs, Cell Culture and Transfection

The stable transfectants of human ACE2 established in 293T cells (i.e., 293T-ACE2 cells) and its parent 293T cell line were purchased from GeneMedi (Shanghai, China; cat. no. GM-SC-293T-hACE2-01) and Merck KGaA (Darmstadt, Germany; cat. no. 12022001). Full-length SDC1, SDC4 and SDC4 deletion mutants and transfectants, established in K562 cells (ATCC CCL-243), were created as described previously [25]. No His-tags were applied for the SDC constructs. Stable SDC transfectants were selected by measuring SDC expression with flow cytometry using APC-labeled SDC antibodies specific for the respective SDC isoform (all RnD Systems, Minneapolis, MN, USA; SDC1: monoclonal rat IgG1 Clone #359103, cat. no. FAB2780A; SDC2: monoclonal rat IgG2B Clone #305515, cat. no. FAB2965A [54,55,56]; SDC3: polyclonal goat IgG, cat. no. FAB3539A [54,57]; SDC4: monoclonal rat IgG2a clone #336304, cat. no. FAB29181A [39,40]), along with respective isotype controls (all RnD Systems; rat IgG1 APC-conjugated isotype control, cat. no. IC005A; rat IgG2B APC-conjugated isotype control, cat. no. IC013A; goat IgG APC-conjugated antibody, cat. no. IC108A; rat IgG2A APC isotype control, cat. no. IC006A).

### 4.3. Flow Cytometry Analysis of HS, ACE2 and SDC Expression

HS and SDC expression of the applied cell lines (293T, 293T-ACE2, K562 and SDC transfectants, Caco-2 and Calu-3) were measured with flow cytometry by using anti-human HS antibody (10E4 epitope; Amsbio, Abingdon, UK; with Alexa Fluor [AF] 647-labeled secondary anti-mouse IgM and respective isotype control: Thermo Fisher Scientific, Waltham, MA, USA, cat. no. 02-6800) and APC-labeled SDC1 or SDC4 antibodies as described previously [39,40]. SDC1 and SDC4 transfectants with almost equal amounts of HS expression were selected for further uptake studies [39,40]. ACE2 expression was measured with human ACE2 AF 647-conjugated antibody (RnD Systems, Minneapolis, MN, USA, cat. no. FAB9332R) and respective isotype control (mouse IgG2A AF 647-conjugated isotype control, RnD Systems, cat. no. IC003R), according to the manufacturer’s protocol.

### 4.4. Establishment of ACE2 or SDC4 KD Cell Lines

SDC4 knockdown in Calu-3 cells was performed using a lentiviral vector system specific to human SDC4 shRNA (cat. no. sc-41400-SH, sc-36588), according to the manufacturer’s protocol (Santa Cruz Biotechnology, Inc., Dallas, TX, USA). Stable KD cells were selected in 2 mg G418 and sorted using imaging flow cytometry (Amnis FlowSight, Luminex Corporation, Austin, TX, USA) with APC-conjugated anti-SDC4 antibody (RnD Systems, Minneapolis, MN, USA, cat. no. FAB29181A) and respective isotype control (rat IgG2A APC isotype control, RnD Systems, cat. no. IC006A). The cellular expression of SDC4 following knockdown was also determined with Western blotting as described previously [25]. A chemiluminescence detection reagent (Luminata Crescendo Western Blotting HRP Reagents) was used for protein visualization, and the signal was detected with UVITEC Alliance Q9 Advanced imager (Uvitec Ltd., Cambridge, UK). β-Tubulin (mouse monoclonal, Santa Cruz Biotechnology Inc., Dallas, TX, USA, cat. no. sc-5274) was used as a loading control.

### 4.5. Pseudovirus (PSV) Studies

WT K562, SDC1 and SDC4 transfectants (created in K562 cells), and Calu-3 cells (WT and SDC4 KD) were seeded in 24-well plates with 1 × 10^5^ cells/well. After 24 h of culture, cells were treated with 2 × 10^6^ transducing units of SARS-CoV-2 PSV-RFP (WT PSV) or its Delta variant (Delta PSV), according to the manufacturer’s instructions (GeneMedi, Shanghai, China). After 72 h of incubation, RFP expression of PSV-treated cells was assessed with imaging flow cytometry (Amnis FlowSight, Luminex, Austin, TX, USA).

### 4.6. Flow Cytometry Analysis of Spike Uptake

293T and 293T-ACE2 cells, K562 and its SDC transfectants, along with Calu-3 cells were utilized to quantify the internalization of the spike proteins. Briefly, 3 × 10^5^ cells/mL in DMEM/F12 medium were incubated with either WT or Delta spike (at a concentration of 50 nM) for 4 h at 37 °C. After 4 h of incubation, the cells were washed and trypsinized (with the method described by Nakase et al. [29,30]) to remove the extracellularly attached spike proteins from the cell surface. The cells were then washed, fixed, permeabilized, and blocked with the appropriate serum for 1 h at room temperature. The cells were then treated with fluorescently (either AF 647 or FITC) labeled anti-6x His tag (a-His) antibodies (rabbit poly and monoclonal, Abcam, Cambridge, UK, cat. no. ab1206 and ab237337) for 1 h. The samples were then rinsed three times with PBS containing 1% BSA and 0.1% Triton X-100 and progressed towards flow cytometry. Cellular uptake was then measured with flow cytometry using an Amnis FlowSight imaging flow cytometer (Amnis Corporation, Seattle, WA, USA). A minimum of 10,000 events per sample were analyzed. Appropriate gating in a forward-scatter-against-side-scatter plot was utilized to exclude cellular debris and aggregates. Fluorescence analysis was conducted with the Amnis IDEAS analysis software.

To analyze the efficacy of trypsinization in removing extracellularly attached proteins, some of the cells were preincubated at 0 °C for 1 h as endocytosis stops at such low temperature [58,59]. Then, the cells were treated with fluorescently labeled (FITC or AF 647) a-His for 4 h at 0 °C. After incubation with the a-His, the cells were washed, trypsinized as described above and cellular fluorescence was then measured with flow cytometry as described above.

To examine the influence of the fluorescently labeled anti-His tag antibodies, some of the cells were washed, fixed, permeabilized and treated with fluorescently labeled a-His and cellular fluorescence was measured with imaging flow cytometry.

### 4.7. Inhibitor Studies

In the inhibitor studies, some cells were preincubated with DX600 (10 µM; Bachem, Bubendorf, Switzerland) or heparin (200 ug/mL; Sigma, St. Louis, MO, USA) 30 min before spike protein or PSV treatment. The cells were then treated with either the spike proteins or PSVs and processed for the flow cytometric analyses described above.

### 4.8. Cell Viability Measurements

The effect of the applied spike proteins on cell viability was assessed with the EZ4U cell proliferation assay (Biomedica Gmbh, Vienna, Austria, cat. no. BI-5000), according to the instructions of the manufacturer. Absorbance was measured with a BioTek Cytation 3 multimode microplate reader.

### 4.9. Co-Immunoprecipitation Experiments

WT Calu-3 cells, incubated with or without the spike proteins, were processed for co-immunoprecipitation experiments as described previously [39,40]. Briefly, after incubation, the cells were washed twice with ice-cold PBS and treated with a cold Pierce IP lysis buffer. The cells were then scraped off to clean Eppendorf tubes, put on a low-speed rotating shaker for 15 min, and centrifuged at 14,000× *g* for 15 min at 4 °C. The supernatant was then transferred to new tubes and combined with 5 µg human SDC4 antibody (RnD System, UK; cat. no. AF2918). The antigen sample/SDC antibody mixture was then incubated overnight at 4 °C with mixing. The antigen sample/antibody mixture was then added to a 1.5 mL microcentrifuge tube containing pre-washed Pierce Protein A/G Magnetic Beads (Thermo Fisher Scientific, Waltham, MA, USA). After incubation at room temperature for 1 h with mixing, the beads were then collected with a MagJET Separation Rack magnetic stand (Thermo Fisher Scientific, Waltham, MA, USA), and supernatants were discarded. To elute the antigen, 100 µL of SDS-PAGE reducing sample buffer was added to the tubes. The samples were heated at 96 °C for 10 min in 1% SDS, and the samples were transferred to SDS-PAGE [39,40]. The samples were then immunoblotted onto PVDF membranes, and the proteins were detected with specific antibodies as described above. Image acquisition was conducted with UVITEC Alliance Q9 Advanced imaging platform.

### 4.10. Statistical Analysis

Results are expressed as means + standard error of the mean (SEM). Differences between experimental groups were evaluated by using one-way analysis of variance (ANOVA). Values of *p* < 0.05 were accepted as significant [39,40]. During imaging flow cytometry, the bright detail similarity (BDS) feature of the Amnis IDEAS software was used to measure colocalization between two signals [60]. A BDS score of 2 or greater represents a high degree of overlap.

## Data Availability

Data are contained within the article or Supplementary Materials.

## Supplementary Materials

The following are available online at https://www.mdpi.com/article/10.3390/ijms23020796/s1.
