# Supplementary material for: Syndecan-4 Is a Key Facilitator of the SARS-CoV-2 Delta Variant’s Superior Transmission
Source: Int J Mol Sci. 2022 Jan 12;23(2):796. doi: 10.3390/ijms23020796 (PMC8775852; doi:10.3390/ijms23020796)
Supplement: Supplementary file 1 [file ijms-23-00796-s001.zip › ijms-1535806-supple-revised2.pdf]

Supplementary Materials: Syndecan-4 is a key facilitator of the SARS-CoV-2 Delta variant's superior transmission

Anett Hudák [1], Gábor Veres [1], Annamária Letoha [2], László Szilák [1], and Tamás Letoha [1,*]

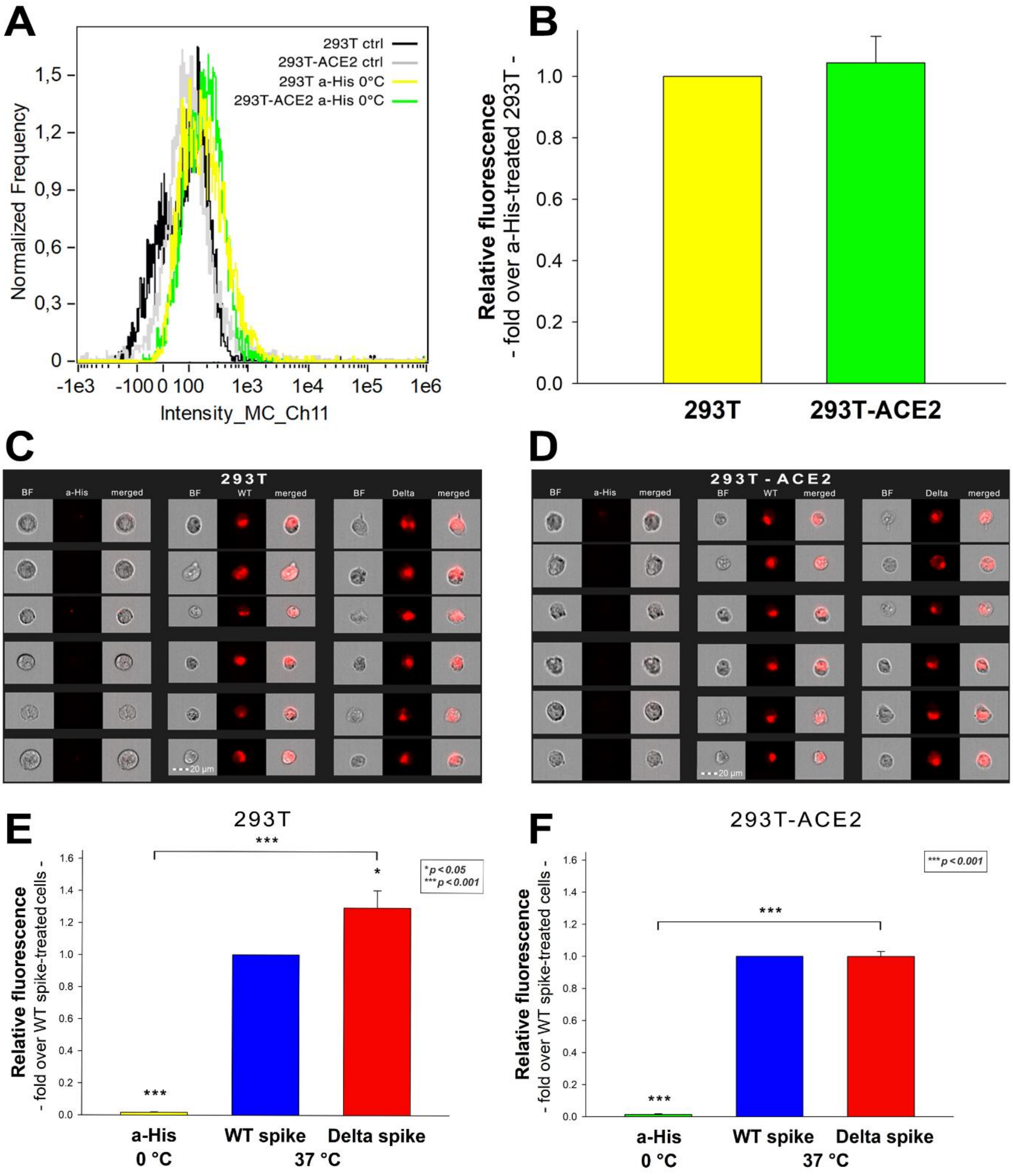

Figure S1. Control studies with 293T and 293T-ACE2 cells treated with AF 647-labeled anti-6x His tag antibody (a-His) at 0 °C. 293T and 293T-ACE2 cells preincubated at 0 °C for 1 h were then treated with a-His for 4 h at 0 °C. After incubation with a-His, the cells were washed, trypsinized and cellular fluorescence was then measured with flow cytometry. **(A)** Flow cytometry histograms showing intracellular fluorescence of a-His-treated and untreated control cells. (**B**) Detected fluorescence intensities normalized to a-His-treated 293T cells as standards. The bars represent the mean + SEM of nine independent experiments. Statistical significance vs. standards was assessed with ANOVA. Compared to standards, a-His-treated 293T-ACE2 cells did not exhibit statistically significant differences in cellular fluorescence. (**C**,**D**) Cellular images of 293T (**C**) and 293T-ACE2 (**D**) cells treated with a-His (at 0 °C) or either of the spike proteins (WT or Delta spike) at 37 °C, as acquired with imaging flow cytometry. (**E**,**F**) Detected fluorescence intensities were normalized to WT spike-treated cells as standards. The bars represent the mean + SEM of nine independent experiments. Statistical significance vs. standards was assessed with ANOVA. **p < 0.05; ***p < 0.001.*

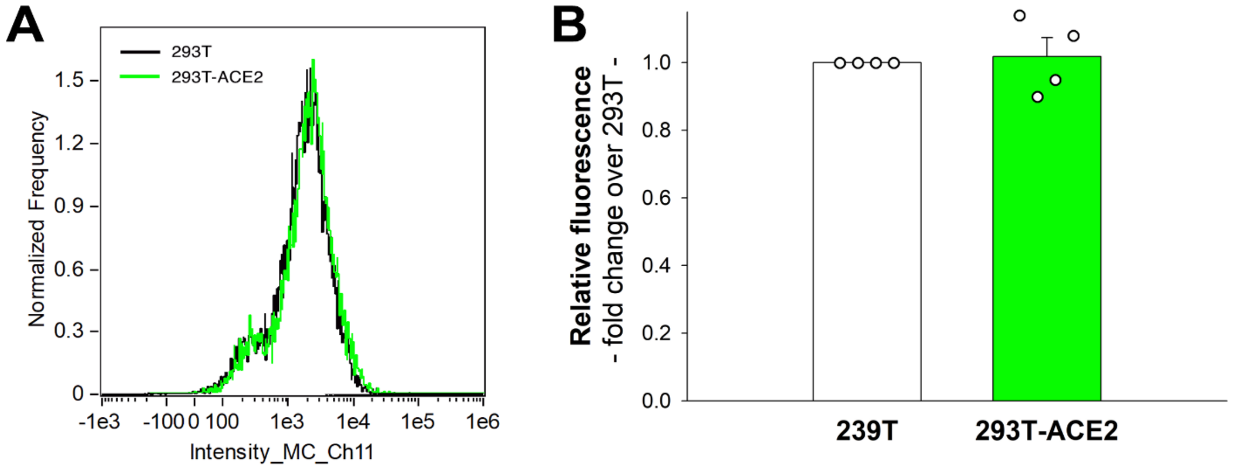

Figure S2. Control studies with permeabilized and a-His-treated and 293T and 293T-ACE2 cells. The cells were trypsinized, fixed, permeabilized and treated with the Alexa647-labeled anti-6X His tag antibody (a-His). Cellular fluorescence was then measured with flow cytometry. **(A)** Flow cytometry histograms showing intracellular fluorescence of anti-His tag antibody-treated, permeabilized cells. (**B**) Detected fluorescence intensities of a-His-treated, permeabilized cells were normalized to a-His-treated 293T cells as standards. The bars represent the mean ± SEM of four independent experiments. Statistical significance vs. standards was assessed with ANOVA. Compared to standards, a-His-treated, permeabilized 293T-ACE2 cells did not exhibit statistically significant differences in cellular fluorescence.

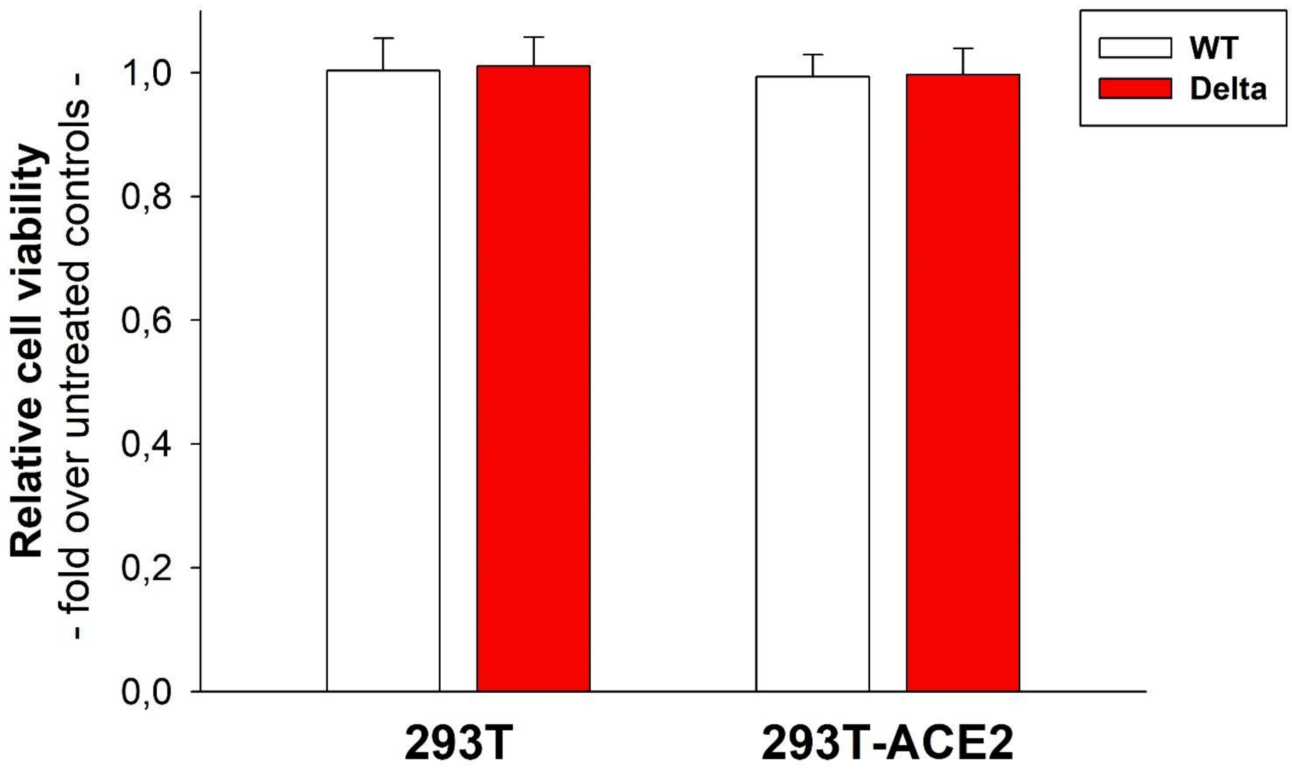

Figure S3. WT and Delta spike proteins do not affect the cellular viability of 293T and 293T-ACE2 cells. 293T and 293T-ACE2 cells were incubated with either the WT or the Delta spike proteins for 4 h at a concentration of 50 nM. Cellular viability was then measured with EZ4U assay and detected measures were then normalized to untreated cells as controls. The bars represent the mean + SEM of three independent experiments. Statistical significance vs. controls was assessed with ANOVA. Compared to controls, no statistically significant differences were detected in the viability of spike-treated cells.

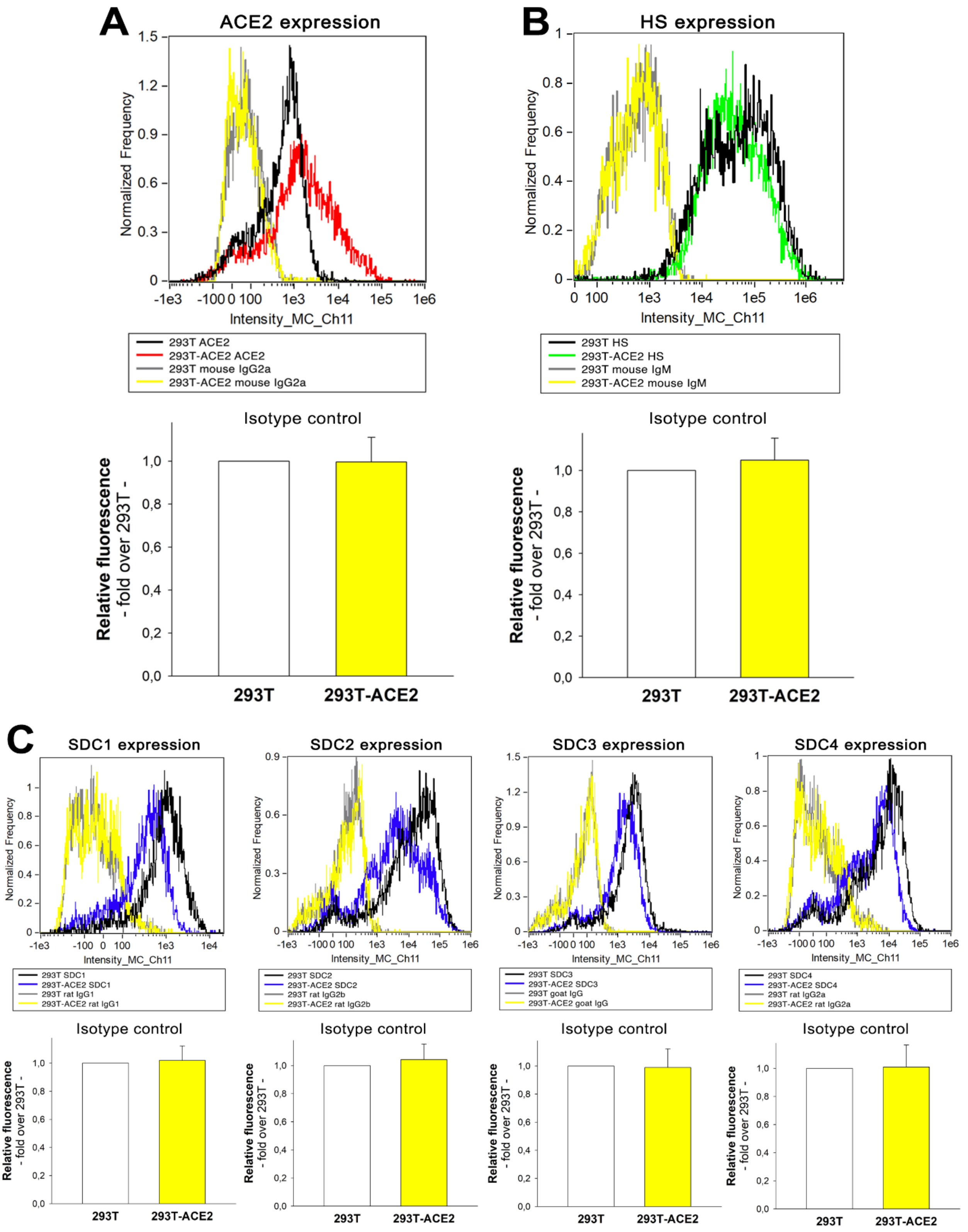

Figure S4. Control studies with 293T and 293T-ACE2 cells treated with ACE2, HS or SDC antibodies and respective isotype controls. The cells were treated with the appropriate antibodies and respective isotype controls. Cellular fluorescence was then measured with flow cytometry. **(A-C)** Flow cytometry histograms showing cellular fluorescence of cells treated with anti-ACE2 (**A**), anti-HS (**B**), anti-SDC (**C**) antibodies or respective isotype controls. Detected fluorescence intensities of isotype control-treated cells were normalized to isotype control-treated 293T cells as standards. The bars represent the mean + SEM of three independent experiments. Statistical significance vs. standards was assessed with ANOVA. Analyses did not exhibit statistically significant differences in cellular fluorescence of isotype-treated cells.

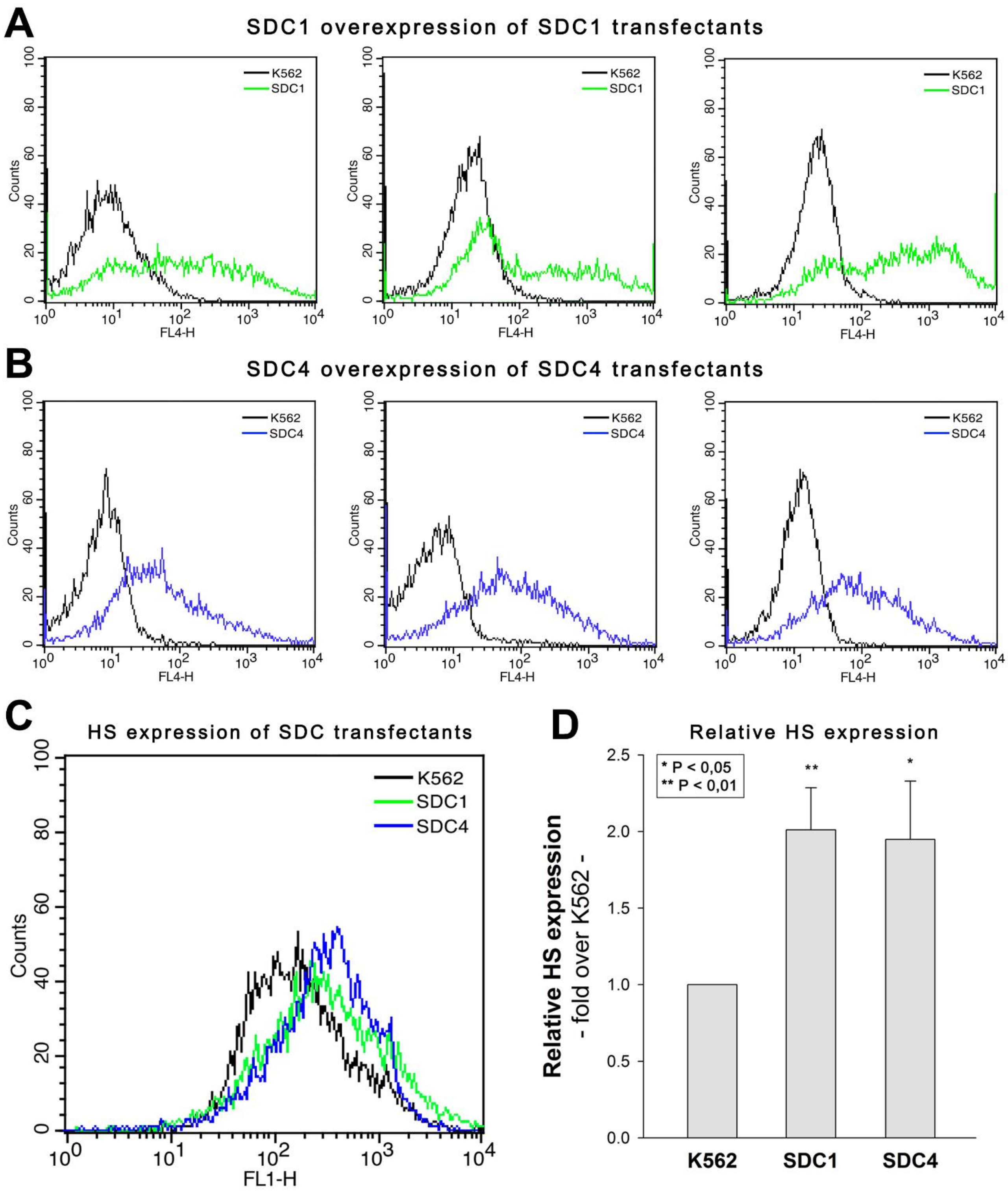

Figure S5. Relative HS expression of SDC1 and SDC4 transfectants. (**A,B**) Stable SDC transfectants created in wild-type (WT) K562 cells were selected by measuring SDC1 and SDC4 expression with flow cytometry (Becton Dickinson FACScan) using APC-labeled anti-SDC antibodies specific for each SDC isoform. Representative flow cytometry histograms showing the SDC1 (**A**) and SDC4 (**B**) expression of SDC transfectants and WT K562 cells. (**C**) Flow cytometry histograms showing HS expression of SDC transfectants and WT K562 cells. The HS expression of SDC transfectants and WT K562 cells was measured by flow cytometry (Becton Dickinson FACScan) using anti-HS antibody. SDC transfectants with a similar amount of HS expression were selected and applied for further studies. (**D**) Detected HS levels were normalized to WT K562 cells as standards. The bars represent the mean + SEM of ten independent experiments. Statistical significance vs. WT K562 cells (standards) was assessed with analysis of variance (ANOVA). *$p<0.05$; **$p<0.01$.*

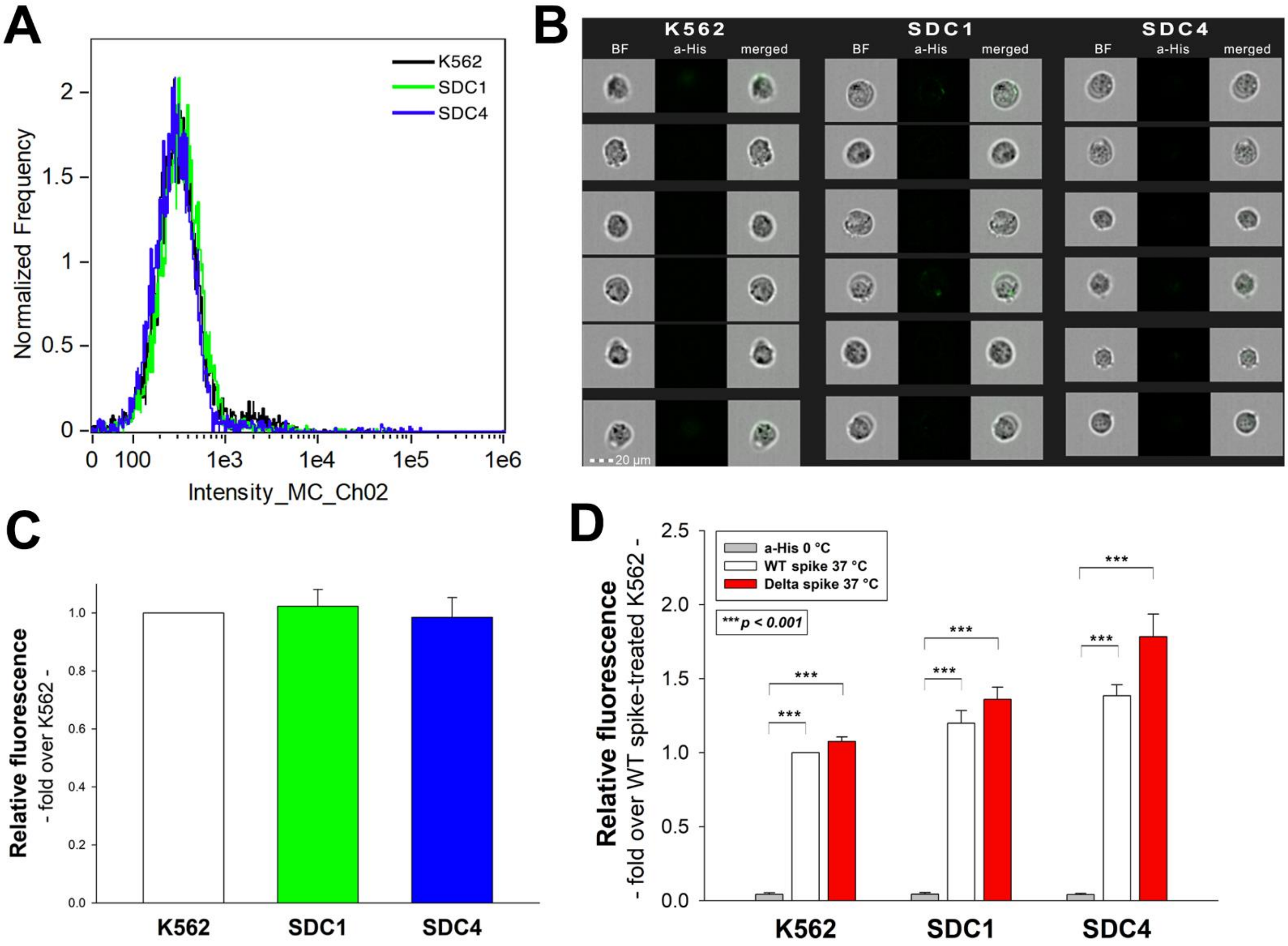

Figure S6. Control studies with WT K562 cells and SDC transfectants (SDC1 and SDC4) treated with FITC-labeled anti-6x His tag antibody (a-His) at 0 °C. WT K562 cells and SDC transfectants preincubated at 0 °C for 1 h were then treated with a-His for 4 h at 0 °C. After incubation with the antibodies, the cells were washed, trypsinized and cellular fluorescence was then measured with flow cytometry. (**A**) Flow cytometry histograms showing intracellular fluorescence of a-His-treated cells. (**B**) Cellular images of a-His-treated K562 cells and SDC transfectants, as acquired with imaging flow cytometry. (**C**) Detected fluorescence intensities normalized to a-His-treated WT K562 cells as standards. The bars represent the mean + SEM of six independent experiments. Statistical significance vs. standards was assessed with ANOVA. No statistically significant differences were detected in the cellular fluorescence of a-His-treated cells. (**D**) Detected fluorescence intensities normalized to WT K562 cells treated with WT spike (at 37 °C) as standards. The bars represent the mean + SEM of six independent experiments. Statistical significance vs. standards was assessed with ANOVA. ***$p < 0.001$.

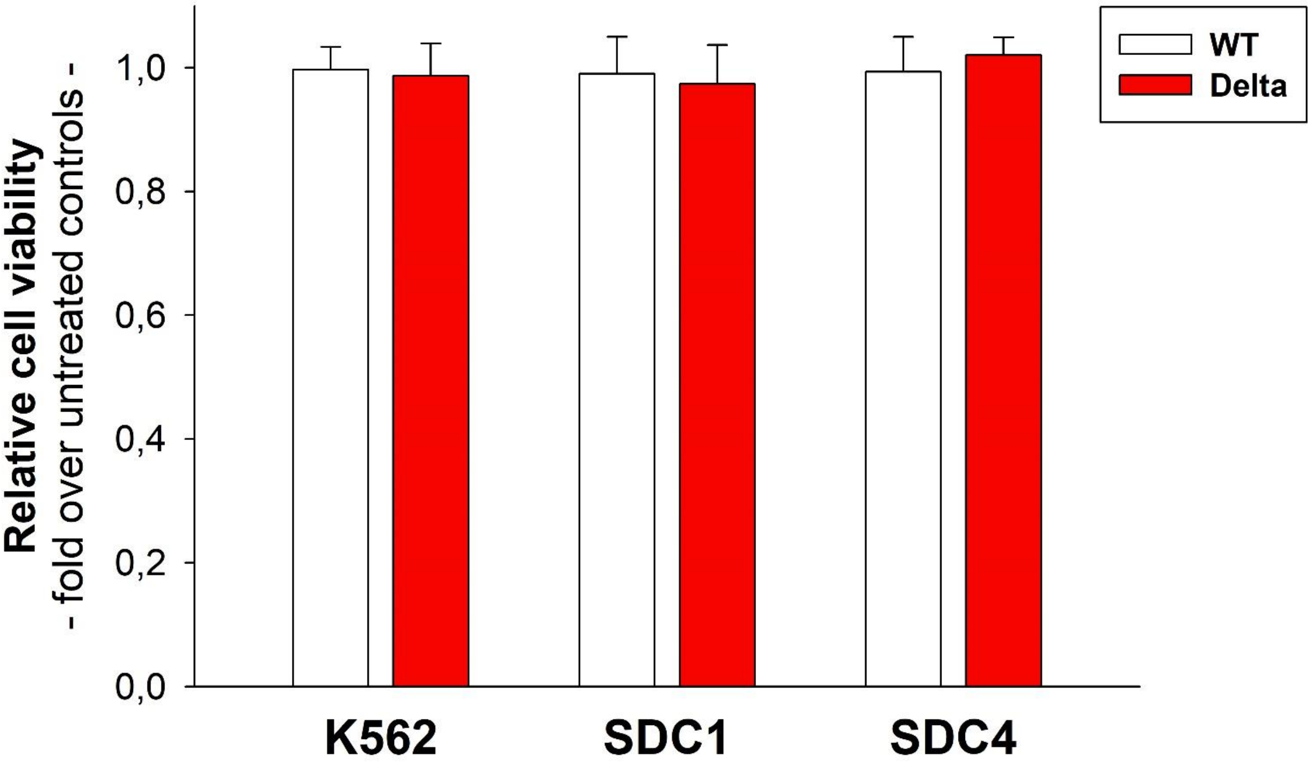

Figure S7. WT and Delta spike proteins do not affect the cellular viability of WT K562 cells and SDC1 and SDC4 transfectants. WT K562 cells, SDC1 and SDC4 were incubated with either the WT or the Delta spike proteins for 4 h at a concentration of 50 nM. Cellular viability was then measured with EZ4U assay and detected measures were then normalized to untreated cells as controls. The bars represent the mean ± SEM of three independent experiments. Statistical significance vs. controls was assessed with ANOVA. Compared to controls, no statistically significant differences were detected in the viability of spike-treated cells.

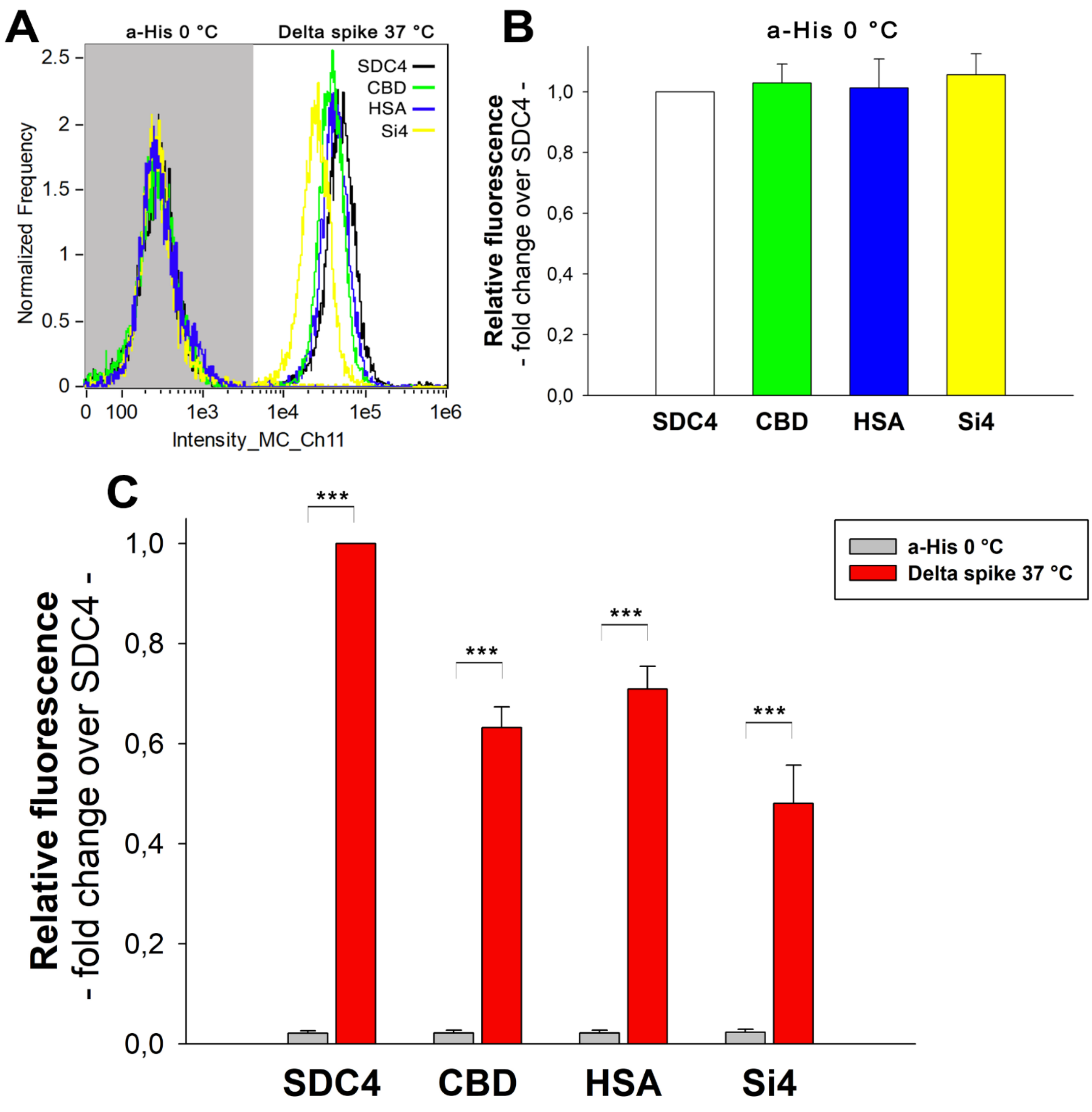

Figure S8. Control studies with SDC4 mutants treated with AF 647-labeled anti-6x His tag antibody (a-His) at 0 °C. SDC4 mutants preincubated at 0 °C for 1 h were then treated with a-His for 4 h at 0 °C. After incubation with the antibodies, the cells were washed, trypsinized and cellular fluorescence was then measured with flow cytometry. (**A**) Flow cytometry histograms showing intracellular fluorescence of SDC4 mutants treated with a-His-treated at 0 °C and those treated with the Delta spike protein at 37 °C. (**B**) Detected fluorescence intensities were normalized to a-His-treated WT SDC4 transfectants as standards. The bars represent the mean + SEM of four independent experiments. Statistical significance vs. standards was assessed with ANOVA. No statistically significant differences were detected. (**C**) Detected fluorescence intensities normalized to WT SDC4 transfectants treated with the Delta spike protein (at 37 °C) as standards. The bars represent the mean + SEM of four independent experiments. Statistical significance was assessed with ANOVA. ***$p < 0.001$.

Supplementary Table S1. ACE2 and SDC expression of Caco-2 and HEK cells as presented in the Human Protein Atlas.

	**Caco-2**	**HEK**
ACE2	0	0.1
SDC1	3.7	1.6 [1]
SDC2	3	6.9
SDC3	0.3	1.4
SDC4	35.1	1.1

[1] Overview of RNA expression levels in Caco-2 and HEK cells analyzed in the Human Protein Atlas. The RNA-sequencing results generated in the HPA are reported as normalized NX values. In the Human Protein Atlas, a NX value of 1.0 is defined as a threshhold for the corresponding protein expression.

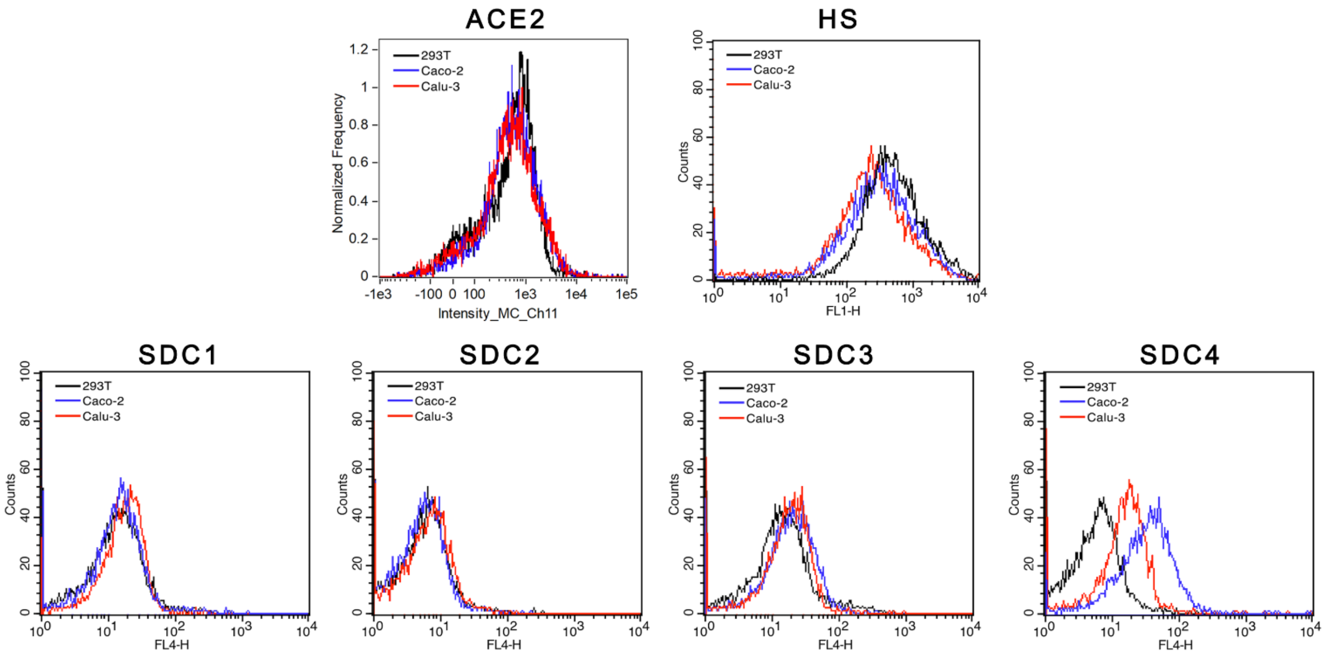

Figure S9. Representative flow cytometry histograms showing the ACE2, HS and SDC expression levels of 293T, Caco-2 and Calu-3 cells as detected with flow cytometry using fluorescently labeled specific antibodies.

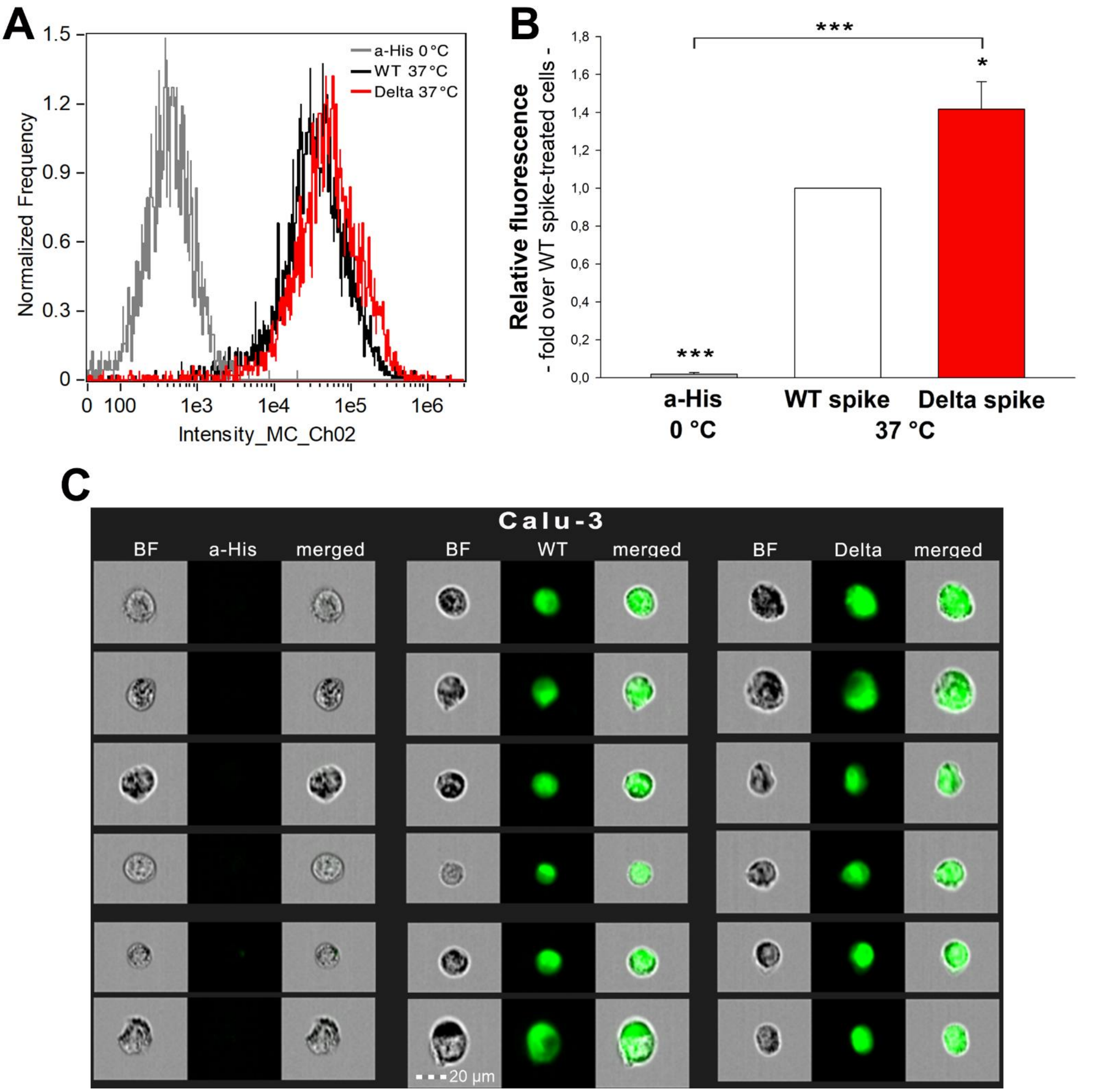

Figure S10. Control studies with WT Calu-3 cells treated with FITC-labeled anti-6x His tag antibody (a-His) at 0 °C. WT Calu-3 cells preincubated at 0 °C for 1 h were then treated with a-His for 4h at 0 °C. After incubation with the antibodies, the cells were washed, trypsinized and cellular fluorescence was then measured with flow cytometry. (**A**) Flow cytometry histograms showing intracellular fluorescence of WT Calu-3 treated with a-His-treated at 0 °C or any of the spike proteins (WT or Delta) at 37°C. (**B**) Detected fluorescence intensities normalized to WT Calu-3 transfectants treated with the WT spike protein (at 37 °C) as standards. The bars represent the mean + SEM of four independent experiments. Statistical significance was assessed with ANOVA. **p < 0.001; ***p < 0.001.* (**C**) Cellular images of WT Calu-3 cells treated with either the a-His or any of the spike proteins (WT or Delta), as acquired with imaging flow cytometry.

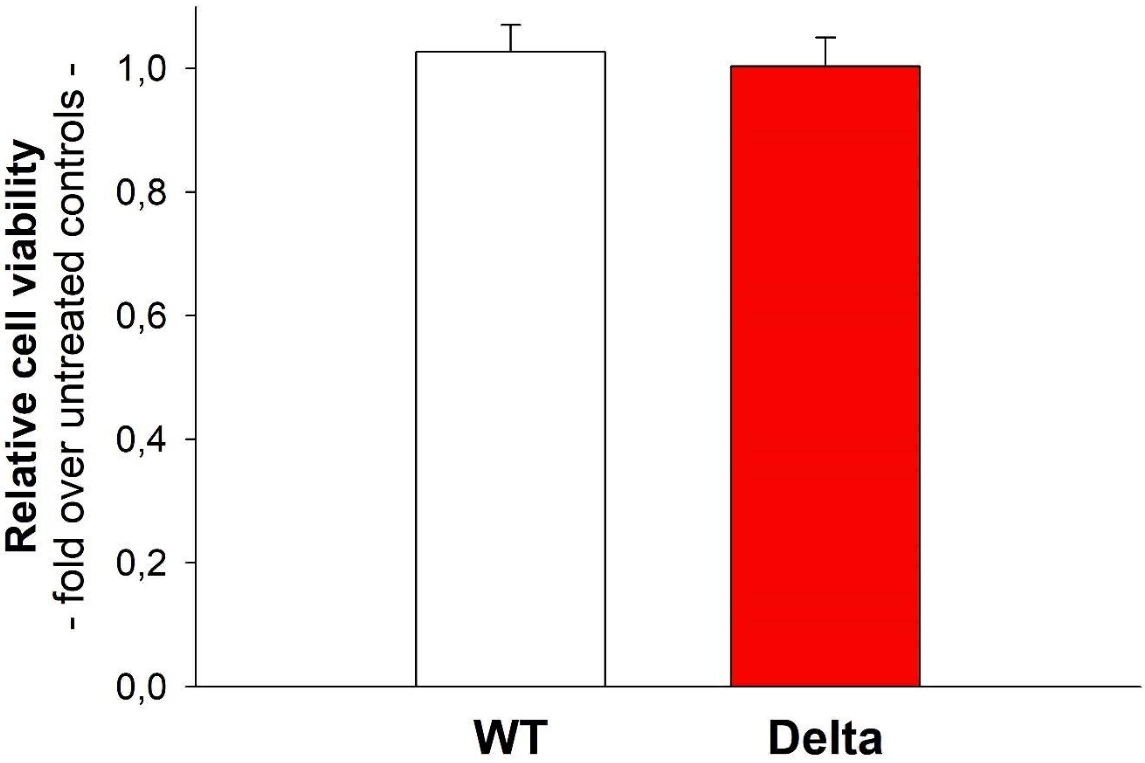

Figure S11. WT and Delta spike proteins do not affect the cellular viability of WT Calu-3 cells. WT Calu-3 cells were incubated with either the WT or the Delta spike proteins for 4 h at a concentration of 50 nM. Cellular viability was then measured with EZ4U assay and detected measures were then normalized to untreated cells as controls. The bars represent the mean ± SEM of three independent experiments. Statistical significance vs. controls was assessed with ANOVA. Compared to controls, no statistically significant differences were detected in the viability of spike-treated cells.

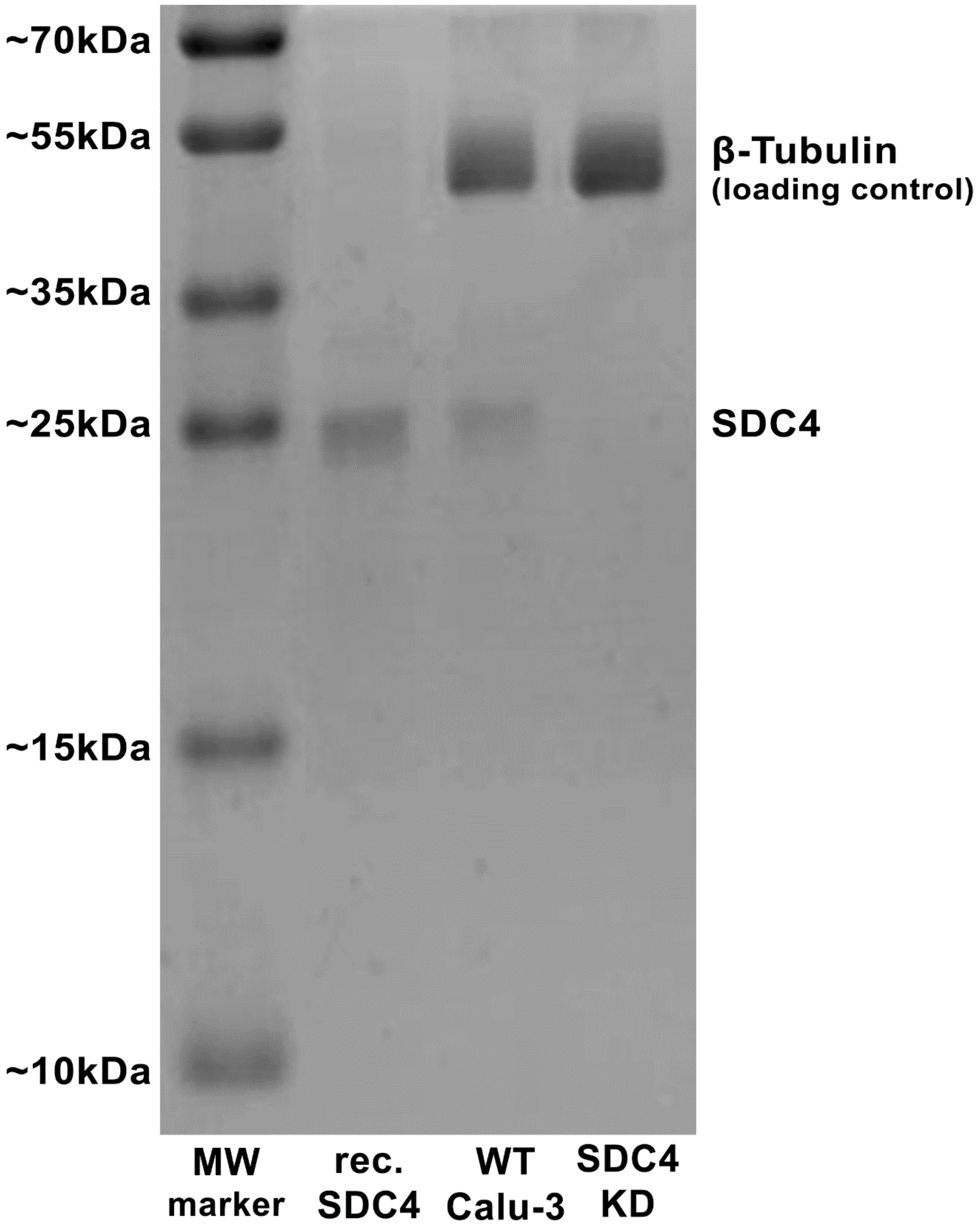

Figure S12. Western blot validation of SDC4 knockdown in Calu-3 cells. SDC4 knockdown (KD) in Calu-3 cells was performed using a lentiviral vector system specific to human SDC4 shRNA. Stable KD cells were selected in 2 mg G418 and sorted using imaging flow cytometry (Amnis FlowSight) with APC-conjugated anti-SDC4 antibodies. Cellular expression of SDC4 following knockdown was also determined with Western blotting. WT Calu-3 and SDC4 shRNA-treated cells were grown in 24-well plates for 24 h, then the medium was removed and the cells were washed (with PBS) and lysed in RIPA buffer. Protein concentrations were measured with spectrophotometer (Metertech UV/VIS). Equal amounts of protein from cell lysates were then subjected to SDS-PAGE on 7,5%–12,5% gradient gels and electroblotted onto PVDF membranes using the Mini Wide Vertical Electrophoresis gel system (Cleaver Scientific). The membranes were blocked in TBST with 5% dry milk, washed, incubated with anti-SDC4 antibodies diluted in TBST with 1% dry milk for 2 h, and then incubated with HRP-conjugated secondary antibodies. Chemiluminescence detection reagent was used for protein visualization and the signal was detected with UVITEC Alliance Q9 Advanced Imager. β-Tubulin was used as a loading control.

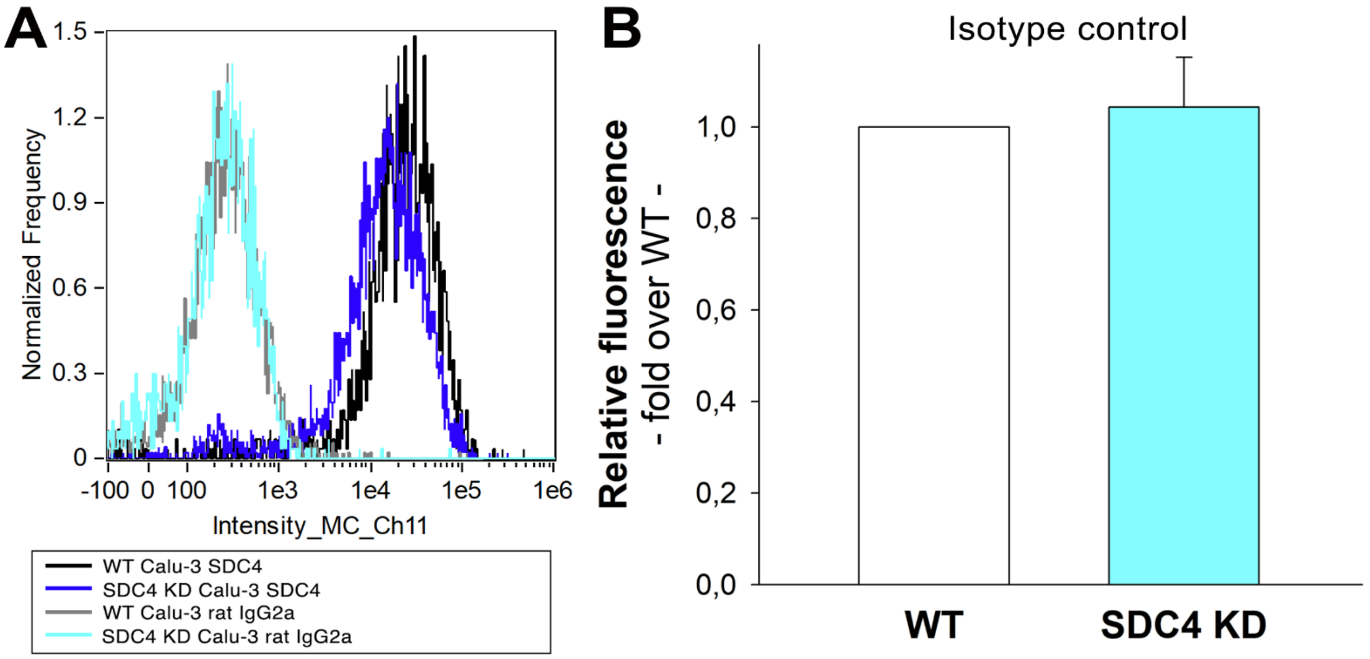

Figure S13. Control studies with WT or SDC4 KD Calu-3 cells treated with APC-labeled SDC4 antibody or the respective isotype control. WT or SDC4 KD Calu-3 cells were treated with APC-labeled SDC4 antibody or the respective isotype control for 1 h. After incubation with the antibodies, the cells were washed and cellular fluorescence was then measured with flow cytometry. (**A**) Flow cytometry histograms showing cellular fluorescence of WT or SDC4 KD Calu-3 cells treated with a-SDC4 antibody and respective isotype control. (**B**) Detected fluorescence intensities normalized to WT Calu-3 cells treated with the isotype control. The bars represent the mean + SEM of three independent experiments. Statistical significance vs. standards was assessed with ANOVA. No statistical significance was detected.
